# An integrated Bayesian analysis of LOH and copy number data

**DOI:** 10.1186/1471-2105-11-321

**Published:** 2010-06-15

**Authors:** Paola MV Rancoita, Marcus Hutter, Francesco Bertoni, Ivo Kwee

**Affiliations:** 1 Istituto Dalle Molle di Studi sull'Intelligenza Artificiale (IDSIA), Galleria 2, 6928 Manno-Lugano, Switzerland; 2 Laboratory of Experimental Oncology, Oncology Institute of Southern Switzerland (IOSI), via Vela 6, 6500 Bellinzona, Switzerland; 3 Dipartimento di Matematica, Università degli Studi di Milano, via Saldini 50, 20137 Milano, Italy; 4 RSISE, ANU and SML, NICTA, Canberra, ACT, 0200, Australia

## Abstract

**Background:**

Cancer and other disorders are due to genomic lesions. SNP-microarrays are able to measure simultaneously both genotype and copy number (CN) at several Single Nucleotide Polymorphisms (SNPs) along the genome. CN is defined as the number of DNA copies, and the normal is two, since we have two copies of each chromosome. The genotype of a SNP is the status given by the nucleotides (alleles) which are present on the two copies of DNA. It is defined homozygous or heterozygous if the two alleles are the same or if they differ, respectively. Loss of heterozygosity (LOH) is the loss of the heterozygous status due to genomic events.

Combining CN and LOH data, it is possible to better identify different types of genomic aberrations. For example, a long sequence of homozygous SNPs might be caused by either the physical loss of one copy or a uniparental disomy event (UPD), i.e. each SNP has two identical nucleotides both derived from only one parent. In this situation, the knowledge of the CN can help in distinguishing between these two events.

**Results:**

To better identify genomic aberrations, we propose a method (called gBPCR) which infers the type of aberration occurred, taking into account all the possible influence in the microarray detection of the homozygosity status of the SNPs, resulting from an altered CN level. Namely, we model the distributions of the detected genotype, given a specific genomic alteration and we estimate the parameters involved on public reference datasets. The estimation is performed similarly to the modified Bayesian Piecewise Constant Regression, but with improved estimators for the detection of the breakpoints.

Using artificial and real data, we evaluate the quality of the estimation of gBPCR and we also show that it outperforms other well-known methods for LOH estimation.

**Conclusions:**

We propose a method (gBPCR) for the estimation of both LOH and CN aberrations, improving their estimation by integrating both types of data and accounting for their relationships. Moreover, gBPCR performed very well in comparison with other methods for LOH estimation and the estimated CN lesions on real data have been validated with another technique.

## Background

Although most of the human genome is identical among individuals, there are about 10 million single nucleotide polymorphisms (SNPs) which distinguish us [1]. SNPs are single base-pair loci where the nucleotides can assume two possible values (called **alleles**) among the four bases (thymine, adenine, cytosine, guanine). In general, since we have two copies of each chromosome, the **genotype **at any SNP can be: *AA*, *BB *or *AB*, where *A *and *B *represent the two alleles. Moreover, a SNP can be classified as **homozygous **(i.e., *AA *or *BB*) or **heterozygous **(i.e., *AB*), whether or not its genotype consists of two equal alleles. Cancer and several human diseases are caused by genomic aberrations, which can affect the homozygous status and/or the DNA copy number (the normal copy number, CN, is two since we have two copies of each chromosome, except for the chromosomes X and Y). The former type of aberrations is often displayed by unusual long stretches of homozygous SNPs, called **loss of heterozygosity **(LOH) region. The latter type of aberrations consists in genomic regions with DNA copy number different from two.

In general, LOH can arise by several mechanisms, such as deletions and germ-line or somatic recombinations. When the LOH occurs without a change in copy number, it is referred to as **copy-neutral LOH **(or sometimes **run of homozygosity**, **ROH**). In the past, copy-neutral LOH regions were usually explained as a consequence of an uniparental disomy event (UPD), see [2]. Recently, long homozygous segments have also been detected in genomes of normal individuals, supporting the hypothesis that some copy-neutral LOH segments might represent autozygosity (see, for example, [3-5]). In the literature, it has been shown a relationship between some tumors and both types of aberrant events (see, for example, [6-8]).

Uniparental disomy (UPD) occurs when both homologues of a part of a chromosome are inherited from only one parent. It can be divided in: uniparental isodisomy (when the two copies are two replicates of one homologue of one parent) and the uniparental heterodisomy (when both homologues are inherited from the same parent). Because of meiotic recombination, a mixture of both events is also possible, and similar events can also happen during the mitosis. Moreover, in cancer cells, the uniparental isodisomy can also occur when a homologue of a part of a chromosome is lost and the remaining homologue is duplicated. The autozygosity describes a situation where the homologues are identical by descendent (IBD), because they are inherited from a common ancestor. Inbreeding is usually uncommon because of laws and social conventions, but it does occur in small isolated populations.

SNP microarrays are able to measure simultaneously both the DNA copy number and the genotype at each SNP position considered [9]. We call **LOH data **the homozygous status of the SNPs deduced from the genotyping data. By integrating the information given by both LOH and copy number data, we can better identify several types of lesions of the genome (regarding combinations of both DNA copy number and LOH aberrations). For example, when one copy of a chromosomal segment is deleted, we usually detect a long stretch of homozygous SNPs (since the genotype calling algorithm is unable to distinguish between the presence of only one copy and the presence of two equal copies), but the same homozygous status can also occur for other reasons, such as uniparental disomy. In this situation, the knowledge of both types of data can lead to the correct interpretation of the phenomenon, while with only one type of data it would not be possible. Another example is when an amplified genomic segment is present: if one of the two copies of the segment is highly amplified, then even the heterozygous SNPs will be likely detected as homozygous, because the DNA quantity of one allele is much higher than the other one. In this case again, the integration of both types of data is able to better identify the dosage of the DNA aberration.

Many methods have been developed for the estimation of the copy number profile (see, for example, [10-14]) and others for the discovery of LOH regions from the genotyping data, without distinguishing if they are caused by either the loss of one copy or other genomic events like uniparental disomy or autozygosity (see, for example, [15,16]). To the best of our knowledge, only one method integrates these two types of data for the estimation of both copy number aberrations and copy-neutral LOH regions and it uses a hidden Markov model (HMM) [17]. Other statistical procedures use the information regarding both the total and the allelic copy number to infer these kind of lesions (for example, [18-24]) and some of these algorithms are available only for the analysis of data coming from Illumina Beadarrays. Finally, in [25] the authors describe an HMM with the same purpose, which employs the allelic copy number data from a tumor sample and the genotyping data from the matched normal sample.

Here, we propose a method which estimates the copy number changes and the copy-neutral LOH regions at the same time, using both LOH and DNA copy number data. The estimation procedure consists of a Bayesian piecewise constant regression, thus we call our algorithm *genomic Bayesian Piecewise Constant Regression *(gBPCR). Our model is more general than [17], since the latter cannot be applied to data, whose DNA sample come from a mixture of cell populations (which is usually the case for samples of patients affected by cancer). Moreover, the algorithm in [17] needs the specification of some parameters by the user and is sensitive to their values.

Our method was implemented in R and is freely available at http://www.idsia.ch/~paola/gBPCR/ or in Additional file 1. Furthermore, an R package will be soon available.

## Methods

Because of the complexity of the biological model, we first describe a preliminary simplified model (called Model 1), which only estimates the copy number events exploiting the relationship between copy number and LOH data. Therefore, it does not identify copy-neutral LOH regions (called IBD/UPD regions), which are due to events like uniparental disomy, and it does not distinguish the normal regions from the gained one (because we suppose that the capability of detection of the homozygous status is the same in these two types of regions). In the subsequent subsections, we add to the model the detection of copy-neutral LOH regions (Model 2) and of gained ones (Model 3). Therefore, the explanation is structured in the following way:

• Model 1: relationship between LOH and copy number data to detect copy number changes (apart from the gained regions);

• Model 2: addition to Model 1 of the IBD/UPD region detection (i.e. determination of copy-neutral LOH regions);

• Model 3: addition to Model 2 of the gained region detection.

Each of the three models is contained in the subsequent. The final model (Model 3) represents our algorithm for the estimation of both copy number changes and copy-neutral LOH regions and we call it *genomic Bayesian Piecewise Constant Regression *(gBPCR).

### Model 1: relationship between LOH and copy number data

Although in nature the copy number is an integer, the raw copy number values detected by the microarray are usually continuous values, due to technical procedures. Moreover, the samples often contain also a percentage of normal cells.

It is common practice to treat copy number data in a log_2_ratio scale (where the ratio is defined with respect to a normal reference dataset) which makes the errors approximately normally distributed. Then, the copy number profile is estimated as a piecewise constant function (i.e. the genome is divided in regions of constant copy number), where the levels assume real values. For the purpose of our model, we estimate this profile by mBPCR, which is a Bayesian piecewise constant regression procedure [14]. It has been shown in [14] that this method outperformed well-known other methods on several datasets.

Commonly, in biomedical/cancer research, after estimating the log_2_ratio profile, the copy number aberrations are defined as those regions with values outside an interval around zero (notice that, in the log_2_ratio scale, zero represents CN = 2, i.e. a normal copy number). Often, the interval is a statistical confidence interval computed on the basis of the samples of the whole dataset.

In Model 1, our aim is to classify better the copy number changes, trying to reduce the number of false positives, by exploiting the relationship between copy number and LOH data.

#### Mathematical model of the biology mechanism

The aim of Model 1 is to obtain a better estimation of the true underlying copy number events, using both the information given by copy number and LOH data. In a genomic region, a copy number event is defined as a particular class of copy number values. The definition of the categories into which the copy number values are divided will follow from the description of the LOH data.

For the purpose of better identifying the copy number events, we can consider two classes of SNP values: **Heterozygosity **(**Het**) and **Homozygosity **(**Hom**). Thus, the **LOH data **are deduced from the genotyping data, by mapping the genotypes AA and BB into Hom and the genotype AB into Het, for all SNPs. The genotype calling algorithm (e.g. BRLMM [26]) is unable to distinguish between a homozygosity due to the presence of two equal nucleotides or the one due to the loss or high amplification of one of them. Hence, the presence of heterozygosity can ensure that the copy number is normal or gained with a high probability, while the homozygosity can be due to different events. It follows that there are only four relevant classes of copy number events that can be distinguished by looking at the LOH data. Therefore, if we call  the random variable which represents a copy number event at SNP *i*, it can assume only the following values:

•  = 2, when CN > 4 (amplification);

•  = 0, when 1 < CN ≤ 4 (normal or gain);

•  = -1, when CN = 1 (loss);

•  = -2, when CN = 0 (homozygous deletion).

The homozygous deletion corresponds to the loss of both copies of a genomic region. Ideally, the genotype calling algorithm should detect a **NoCall **genotype at the corresponding SNP position (i.e. it should not be able to identify the genotype of the SNP). Although not common, since cancer DNA samples usually contain a mixture of normal and tumor cells (with also different cancer cell subpopulations), the information given by the *NoCall *genotype can be used to better distinguish between a mono-allelic deletion and a bi-allelic (homozygous) deletion.

Therefore, three different LOH variables are present in the model: the true homozygous status in normal cells (**X**^*N*^), the homozygous status in abnormal cells (**X**), which is the consequence of copy number changes (in Model 1 we do not consider other biological events), and the homozygous status detected by the genotype calling algorithm (**Y**). The components of the first two random vectors can assume only values in  = *{Het*, *Hom} *and ^*^ = {∅, *Het*, *Hom*}, respectively, and we suppose that they are independently distributed as Bernoulli random variable. The components of **Y **can assume values in  = {*NoCall*, *Het*, *NHet*} (*NHet *stands for "not heterozygous", since the genotype calling algorithm cannot distinguish between two equal nucleotides, i.e. homozygosity, and the loss of one copy).

**Figure 1:**
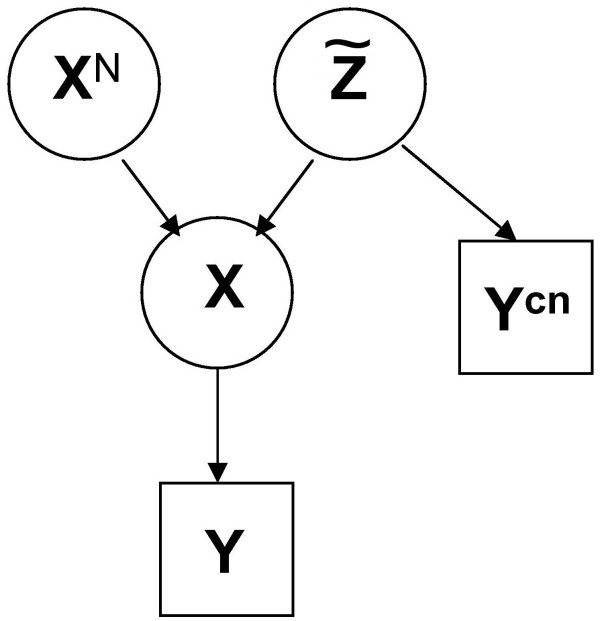
**Scheme of Model 1**. The vector **X **of the homozygous status of all SNPs in abnormal cells is completely determined, given the vector **X**^*N *^of their homozygous status in normal cells and the vector  of their corresponding copy number events. Using this relationship among **X**, **X**^*N *^and , we can estimate , given the observations **Y**^*cn *^and **Y **(respectively, the raw log_2_ratio of the copy number and the homozygous status in abnormal cells detected by the genotype calling algorithm) and by specifying the prior distribution of **X**^*N*^. The observations **Y**^*cn *^are used to defined the prior distribution of  in the Bayesian model.

A summary of the model can be found in Figure 1 and a summary of the notations is in Table 1. Ideally, at each SNP *i*, the homozygous status in abnormal cells *X*_*i *_is completely determined, given the corresponding value in normal cells  and the occurred copy number event , by the following relations:

**Table 1:** Notations.

*Het*	heterozygous
*Hom*	homozygous
*NHet*	not heterozygous (is used when we cannot distinguish between two equal nucleotides, i.e. homozygosity, and the loss of one copy)
	{*Het, Hom*}
^*^	{ø, *Het, Hom*}
	{*Het, NHet, NoCall*}
**X**^*N*^	true genotypes in normal cells ( ∈ )
**X**	true genotypes in abnormal cells (*X*_*i *_∈ ^*^)
**Y**	genotypes detected by the genotype calling algorithm (*Y*_*i *_∈ )
**Y**^*cn*^	raw copy number data
	copy number events/aberrations
	occurrence of copy-neutral LOH (i.e. IBD/UPD event)
	IBD/UPD & copy number aberrations ({ = *w*} = {, = *u*} for some *w, z, u*)
*cn*	all copy number information (both raw data and estimated profile by mBPCR)
**p**	vector of posterior probabilities to be a breakpoint (for all SNP positions)

Nevertheless, the homozygous status of abnormal cells estimated by the genotype calling algorithm (*Y*_*i*_) is affected by several sources of errors.

#### Hypothesis of the model

The genome of cancer cells can be divided in subregions where the copy number is constant. Since we divided the copy number values in four classes (i.e. the copy number events), we can also consider regions with the same copy number event.

Let us consider a genomic region where the microarray measures the DNA copy number and the genotype at *n *SNP loci. Then, from the previous discussion, the vector of the copy number events at all positions  can be seen as a piecewise constant function. This function consists of *k*_0 _intervals with the same copy number event and with boundaries  so that , for all *p *= 1, ..., *k*_0_. To estimate this function we use a Bayesian piecewise constant regression method, which determines the number of segments *k*_0_, the boundaries () and the copy number events .

**Figure 2:**
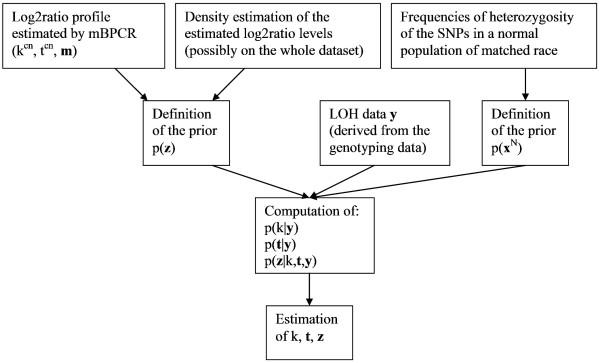
**Scheme of the algorithm corresponding to Model 1**. The graph shows the algorithm for the inference of Model 1. The scheme of gBPCR (i.e. Model 3) can be obtained by substituting *p*(**z**|*k*, **t**, **y**) with *p*(**w**|*k*, **t**, **y**) and by estimating **w** instead of **z**.

For any sample, we assume to have the homozygous status detected by the genotype calling algorithm (**Y**) and the profile of the log_2_ratio of the copy number estimated by mBPCR. The estimated log_2_ratio profile consists of  intervals with boundaries  and levels of the segments  ( is the estimated log_2_ratio in the p^*th *^interval, for ). This estimated profile is used only to define the prior distribution of the random vector **Z **(see Subsubsection "**Z **prior definition"), while the LOH data **Y **are used to infer **Z **(the scheme of the algorithm is in Figure 2). Notice that we do not suppose to know **X**^*N*^, i.e. the homozygous status in normal cells. Moreover, we assume that, given the true value of the homozygous status in normal cells **X**^*N *^and the copy number event **Z **at each position, the LOH data points  are independent, since their values depend only on both noise and genotype detection errors.

The model implies that, given *k*_0 _and **t**^0^, the posterior distribution of  is

and thus, if we condition only with respect to the LOH data **y**, the posterior becomes

where  and  are the domains of *k *and **t**, respectively (they will be defined later).

To specify the model (see Figure 1), we need to define the likelihood, i.e. the conditional distribution of **Y**, given  and **X**^*N*^. To model it, we take into account all the variability that can affect the genotype detection, such as: the polymerase chain reaction (PCR) amplification, the presence of different cancer cell subpopulations or normal cells, and the amplification of only one copy. For example, the probabilities  and  are not zero, because of the error in the genotype detection even in case of a normal DNA sample. The probabilities  and  are related to the detection errors due to the presence of normal cells and/or different types of cancer cell subpopulations, or to PCR amplification errors, while  is related to the errors that can be due to the amplification of only one allele. Also  and  account for the errors that can be due to the presence of cell subpopulations.

The set of conditional probabilities  are considered as parameters of the model. To quantify them, we needed paired normal-cancer samples, since they are related to the probability of detecting a certain homozygous status in a cancer cell, given the corresponding one in a normal cell of the same patient and under some copy number event. Therefore, to compute maximum likelihood estimates of these parameters, we used 13 samples from an available cancer dataset consisting of breast cancer cell lines [27,28] (see Section S.1 in Additional file 2, for further explanations).

To complete the Bayesian model, we need to define the prior distributions of the other random variables. For the parameters *K *and **T**, we consider distributions similar to the ones used in mBPCR [14]:

where  = {1, ..., *k*_max_} and  is a subspace of  such that *t*_0 _= 0, *t*_*k *_= *n *and *t*_*q *_∈ {1, ..., *n *- 1} for all *q *= 1, ..., *k *- 1, in an ordered way and without repetitions.

The prior probabilities of heterozygosity of the SNPs  are the frequencies of heterozygosity computed on the samples of the matched race in the HapMap project [3,29]. They are usually provided by the manufacturer in the documentation related to the microarray used. In Section "Results and Discussion", the microarray mostly employed is the GeneChip Human Mapping 250K NspI (Affymetrix, Santa Clara, CA, USA).

#### Z prior definition

The only prior that we have not yet defined is the one of **Z**. While the estimated levels of the log_2_ratio profile are continuous variables, *Z *classifies the copy number as discrete events. Then, the major problem consists in mapping the continuous values into the discrete values of *Z*, i.e. in defining a partition of the log_2_ratio values such that each interval corresponds to a particular copy number event.

**Figure 3:**
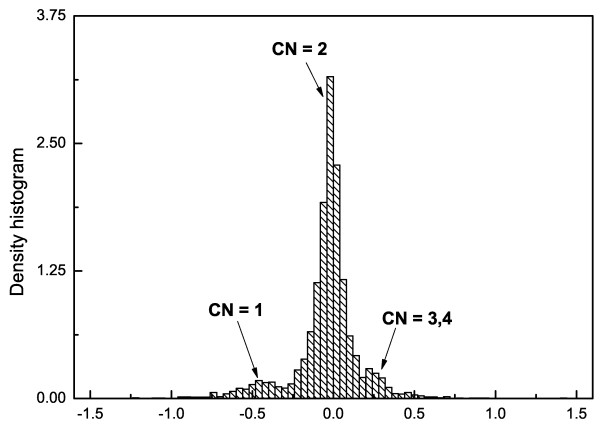
**Example of a histogram of estimated log2ratio levels**. The graph shows the histogram of the mBPCR estimated log2ratio levels of the profiles of 14 HIV lymphoma cell lines in [32].

In the literature, most methods determine a confidence interval around zero (which corresponds to *CN *= 2) and then consider all the log2ratio values above this interval as gains and all values below as losses (see, for example, [30,31]). This method is not suitable in our case, since we want to classify also the events {*CN *= 0} and {*CN *> 4}. Looking at the histogram of the estimated log_2_ratio values (see, for example, in Figure 3 the histogram derived from the 14 HIV lymphoma cell lines in [32]), we can see that they have a multimodal density with peaks corresponding to *CN *= 1, *CN *= 2 and *CN *= {3, 4}. Sometimes, we can even separate the peaks of *CN *= 3 and *CN *= 4. Similarly to [33], we model this density as a mixture of normal distributions (a way to estimate this mixture density can be found in Section S.2 in Additional file 2). Once the parameters of the density are estimated, we can define a function to map the log_2_ratio values into the copy number event values:(1)

where  are, respectively, the estimated mean and variance of the normal distribution corresponding to *CN *= *cn*.

From the definition of *f*_*LOGtoZ*_, for all *p *= 1, ..., , we define the prior distribution of *Z*_*p *_as:

where *cn *represents all copy number information (both raw data and estimated profile by mBPCR) and *M*_*p *_is the random variable representing the log_2_ratio value in the *p*^*th *^segment. From the mBPCR model, given *cn*, *M*_*p *_is normally distributed with mean  and variance  where (, ) are the posterior mean and variance of *M*_*p *_estimated by mBPCR, respectively.

#### The estimation

To estimate the piecewise constant profile of the copy number events, we define the estimators of *k*_0 _(the number of segments) and **t**^0 ^(the boundaries) similarly to the ones in the mBPCR method [14]:(2)(3)

Namely,  corresponds to the  positions which have the highest posterior probability to be a breakpoint. The main differences with respect to mBPCR are in the prior over *K *and in the estimation of *K*. Instead of using a uniform prior and an estimator which minimizes the posterior expected squared error, here we consider a prior similar to 1/*k*^2 ^and an estimator which minimizes the 0-1 error, in order to reduce the false discovery rate (FDR) in case of few segments.

Another difference with respect to mBPCR consists in the level estimation. While in the copy number model the levels were continuous random variables, now they assume categorical values. Hence, they are estimated separately (as before) with the MAP estimator instead of the posterior expected value,(4)

for *p *= 1, ..., , where  and  are any estimate of **t**^0 ^and *k*_0_, respectively. For the computation of all the posterior probabilities involved, we used dynamic programming as described in Section S.3 in Additional file 2.

Let us define **y**_*ij *_= (*y*_*i*+1_, ..., y_j_), representing the LOH data points in the interval [*i *+ 1, *j*], and *K*_*ij *_as the random variable which represents the number of segments in the interval [*i *+ 1, *j*]. Using Bayes' Theorem and the independence of the LOH data points belonging to different segments, the probability in Equation (4), given the LOH data **y**, can be written as,(5)

Therefore, if the boundary estimator misses a clear boundary between  and , then the probability at the denominator of Equation (5) could be zero and thus the level would not be estimated. The best way to prevent this event consists in using a good estimator for the boundaries.

**Figure 4:**
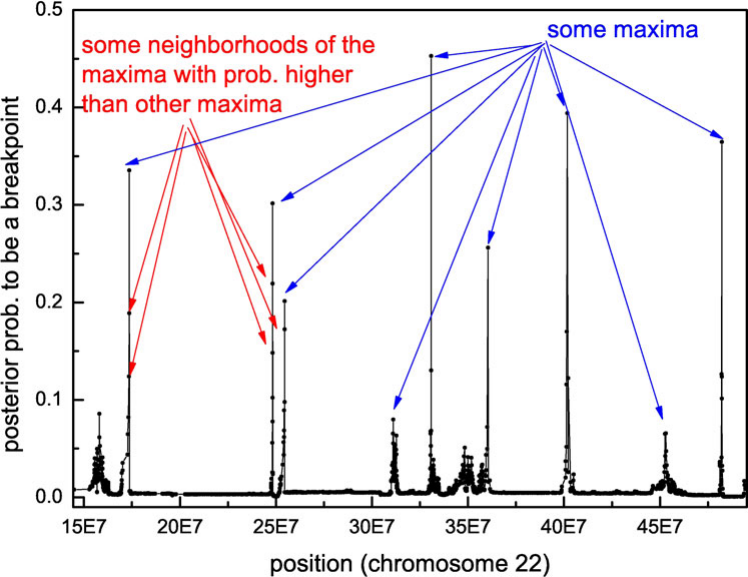
**Example of estimated posterior probabilities to be a breakpoint**. The graph shows, for each probe, the estimated posterior probability to be a breakpoint on a sample of dataset *B*.

Previously, in [14] we found that the boundary estimator  is an estimator with a high sensitivity, but medium FDR. The problem of this estimator is the following. The vector **p **of the posterior probabilities to be a breakpoint at each point of the sample usually represents a multimodal function with maxima at the breakpoint positions, but often in a neighborhood of each maximum there are other points with high probability because of the uncertainty (see Figure 4). Hence, if we take the first  points with the highest probability (according to the definition of ), we could take points in the neighborhood of the higher maxima and not some maxima with a lower probability (see Figure 4). As a consequence, if *k*_0 _was estimated with its exact value then the sensitivity of the  would be lower. In this case, we could lose important breakpoints so that the denominator in Equation (5) would become zero. In practice,  often slightly overestimates *k*_0_, because of the high noise of the data, and thus this phenomenon should not happen, but to prevent even this rare case we searched for a way to improve the estimation of the boundaries.

Since commonly the vector of the posterior probabilities shows clearly the position of the breakpoints in correspondence to the maxima, we estimate the number of the segments and the breakpoints with the number of peaks and the locations of their maxima, respectively (see Section S.4 in Additional file 2). Essentially, the algorithm for the determination of the peaks, after applying a kernel method to reduce the noise of the function, uses two thresholds: one for the determination of the peaks (*thr*_1_) and one for the definition of the values close to zero (*thr*_2_). Therefore, we will denote the corresponding estimators by  and .

In Section "Results and Discussion", we will consider several pairs of thresholds and we will apply the corresponding estimators to simulated data, in order to determine the best paired thresholds and to compare their performance with . We will also compare  with , another boundary estimator described in [14].

### Model 2: addition of the IBD/UPD region detection

LOH data are used in biology not only to better identify regions of loss and amplifications, but, especially, to detect regions of copy-neutral LOH, which can be identified by unusual long stretches of homozygous SNPs, with normal copy number. In Section "Background", we explained that this type of aberrations can be a consequence of UPD (either uniparental isodisomy or heterodisomy) or autozygosity (IBD regions). From the description of these genomic events, it follows that the uniparental isodisomy and the IBD regions can be detected because they appear as a long sequence of homozygous SNPs with a low probability to occur, while the uniparental heterodisomy consists in a sequence of both homozygous and heterozygous SNPs as in a normal condition. Therefore, without the genotypes of the parents, from SNP data we can only detect the uniparental isodisomy (iUPD) and the IBD segments. In the following, we will consider only these two events, referring to them as IBD/UPD events.

Since an IBD/UPD event, by definition, only exists in regions of normal copy number (CN = 2), the only probabilities which are affected by the presence of this event are those involving {*Z *= 0}. Therefore, we define the following sets of conditional probabilities  and , where the variable  indicates if an IBD/UPD event occurred at SNP *i *(if it happened  = 1, otherwise  = 0). We can notice that, given { = 0,  = 0}, the distribution of *Y*_*i *_is equal to the conditional distribution with respect to { = 0} in Model 1, since the latter was modeled with no possibility of an IBD/UPD event. Instead, in case of an IBD/UPD event, we do not need to condition with respect to , since, in case of a somatic iUPD event, the genotype of an iUPD region is independent of the homozygous status of the same region in a normal cell. Otherwise, in case of autozygosity or germ line iUPD, the genotypes of normal and abnormal cells are the same and it makes no sense to condition one to the other.

**Figure 5:**
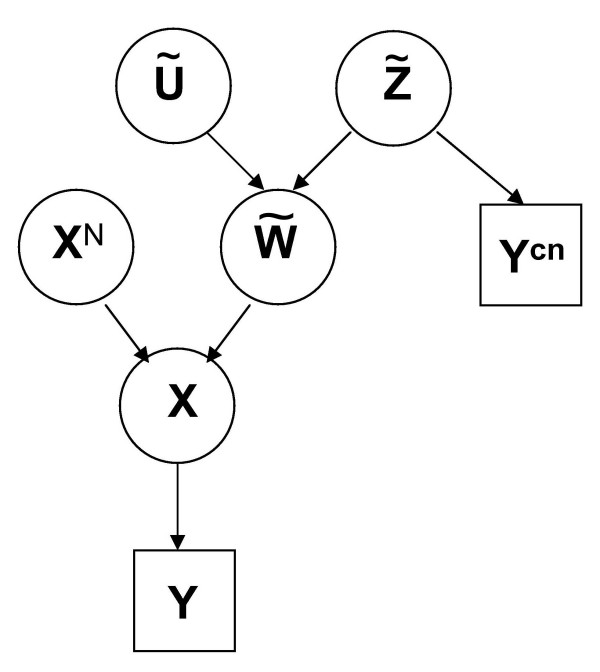
**Scheme of Model 2 and 3**. The vector  of aberration events represents the lesions derived from both IBD/UPD events () and copy number event (), at each SNP position. The vector **X **of the homozygous status of all SNPs in abnormal cells is completely determined, given the vector **X**^*N *^of their homozygous status in normal cells and the vector  of their corresponding aberration events. Using this relationship among **X**, **X**^*N *^and , we can estimate , given the observations **Y**^*cn *^and **Y **(respectively, the raw log_2_ratio of the copy number and the homozygous status in abnormal cells detected by the genotype calling algorithm) and by specifying the prior distributions of  and **X**^*N*^. The observations **Y**^*cn *^are used to defined the prior distribution of  in the Bayesian model.

In the new framework, we define the vector of the aberration events at *n *SNP loci as ; here the aberrations regard both copy number changes and IBD/UPD regions. Each component  of the vector assumes values: -3 ( = 0 and  = 1, i.e. IBD/UPD event), -2 ( = -2, i.e. homozygous deletion), -1( = -1, i.e. loss), 0 ( = 0 and  = 0, i.e. normal state or gain), 2 ( = 2, i.e. high amplification); a graphical representation of the model is given in Figure 5. As previously, we can divide the genome in intervals corresponding to the same aberration event, i.e the profile of the aberrations consists of *k*_0 _intervals, with boundaries , so that , for all *p *= 1, ..., *k*_0_. The estimation procedure is similar to the one of Model 1. The estimators of *k*_0 _and **t**^0 ^are the same and, given  and  (any estimate of *k*_0 _and **t**^0^, respectively), we estimate the aberration events in each interval with their MAP estimators,(6)

for *p *= 1, ..., . Notice that, for *w *= -2, -1, 2, the posterior probability P(*W*_*p *_= *w*|**Y**, , , *cn*) is equal to P(*Z*_*p *_= *w*|**Y**, , , *cn*), while for *w *= -3, 0 we have,(7)(8)

and we assume that P(*U*_*p *_= 1) =: *p*_*upd*_, for all *p *= 1, ..., .

Both  and *p*_*upd *_are parameters of the model. For the maximum likelihood estimation of , we used 11 IBD/UPD regions previously found by us on 5 samples of patients with hairy cell leukemia [34] and on the B-cell lymphoma cell line KARPAS-422. All regions were detected by dChip [16] and their width was between 3 Mb and 100 Mb (covering from 300 to 9800 SNPs), so that they were large enough to be really considered IBD/UPD regions (for further explanations, see Section S.1 in Additional file 2).

#### Values for the parameter p_upd_

We expect the prior probability of an IBD/UPD event to be low. In order to estimate the order of magnitude of this parameter, we considered two studies on IBD regions: [6] and [3]. In the former, they considered as IBD regions only stretches of at least 50 homozygous SNPs (with at maximum 2% of heterozygous) longer than 4 Mb and the platform used was the Affymetrix GeneChip Human Mapping 50K Array. In the latter, a denser microarray was used and the stretches considered were longer than 1 Mb (with at least 50 probes) or longer than 3 Mb. Using the data of the former paper (only the normal samples), we estimated *p*_*upd *_≈ 1.7·10^-3^. Instead, with the data of the latter, we estimated *p*_*upd *_≈ 1.5·10^-3 ^considering all regions greater than 1 Mb, while *p*_*upd *_≈ 1.46·10^-4^, considering only the regions greater than 3 Mb. The differences in the estimated values are due to the different resolution of the technologies used (in fact, in the former the number of SNPs used was 58,960, while in the latter it was 3,107,620). Moreover, the probability depends on the minimum length allowed for these regions. The wider the regions are, the higher is the probability that the regions represent "abnormalities" and the lower becomes the probability of their occurrence (so that *p*_*upd *_is lower). Therefore, in the following applications (see Section "Results and Discussion"), we will use two values: *p*_*upd *_= 10^-3 ^and *p*_*upd *_= 10^-4^.

Another possible way to solve the problem could be to assign a prior distribution to *p*_*upd *_(for example, a uniform distribution over its range) and integrate it out in the equations of the model.

### Model 3: addition of the gained region detection

In the description of Model 1, we explained our assumption that there is no difference in the genotype detection between a normal or gained region. Therefore, in Model 1 (and in Model 2), we defined a single class for the normal or gained regions. But, for the biological studies, it is relevant to distinguish these two copy number events and this distinction is based essentially on the estimated copy number (since there is no difference in the distribution of the detected genotypes, due to the previous discussion). As a consequence, the probability of *Y*_*i *_given a normal (i.e. { = 0}) or gained copy number (i.e. { = 1} = { = 1}) is the same,

We also need to define two distinct prior probabilities for the normal copy number and the gain event. Similarly to its previous definition, for all , the new prior of *Z*_*p *_ is given by,

In the following, Model 3 (which is the complete model) will be called *genomic Bayesian Piecewise Constant Regression *(gBPCR).

### Adjustment of the parameters related to *NoCall*

The probabilities {P(*Y*_*i *_= *NoCall*| = *x*,  = *w*), *x *∈ , *w *= -3, -2, -1, 0, 2} are related to the detection of *NoCall*s under some conditions. Generally, the presence of *NoCall*s is not only due to diffculties of the genotype calling algorithm in the detection of the genotype (technical noise) but also to the noise of the sample because of differences in the quality of extracted DNA. Therefore, we need to adjust the estimated values of these parameters on the basis of the sample noise.

Since usually the *NoCall *rate (i.e. percentage of *NoCall*s in the sample) increases with the noise of the sample, we assume that, given { = *x*,  = *z*}, the probability of detecting a *NoCall *at SNP *i *in sample *s *is proportional to a parameter *p*_*x,z *_(which depends on the technical noise) by a factor *θ*_*s *_(which depends on the sample noise),(9)

If we condition over the values of  and estimate P( = *Het*) = 1/2 for a generic SNP *i *(by considering a uniform distribution over the four possible combinations of alleles AA, AB, BA, BB), we can compute the *NoCall *rate in regions with copy number event *z *in the following way,(10)(11)

Therefore, by applying Equations (9) and (11), for any pair of samples (Sample 1 and 2), we can write the conditional probability of *NoCall*, given { = *x*,  = *z*}, in Sample 1 in terms of the corresponding probability in Sample 2,(12)

In the following, we will denote the sample to estimate with *s *= 1 and the reference sample with *s *= 2.

Using Equation (12), the values of the parameters related to *NoCall *detection are adjusted for Sample 1,

for *z *= -2, -1, 0, 2, where *r*_1_(*z*) and *r*_2_(*z*) are an estimate of the *NoCall *rate in regions with copy number event *z*, for Sample 1 and 2, respectively. By applying Equation (10) with P( = *Het*) = 1/2, *r*_2_(*z*) can be computed from the estimated values of P(*Y*_*i *_= *NoCall*| = *Het*,  = *z*) and P(*Y*_*i *_= *NoCall*| = *Hom*,  = *z*)

for *z *= -2, -1, 0, 2. *r*_1_(*z*) is the frequency of *NoCall *in regions with copy number event *z *of Sample 1, for *z *= -2, -1, 0, 2.

The estimated value of the probability P(*Y*_*i *_= *NoCall*| = -3) is adjusted in a different way. On the reference samples, we found, as expected, that

that is, the *NoCall *rate in IBD/UPD regions is approximately equal to the *NoCall *rate in normal regions. Therefore,

In Section "Results and Discussion", we will compare the estimations resulting from gBPCR with and without the adjustment of these parameters.

## Results and Discussion

In this section, we apply gBPCR to both artificial and real data. First, we compare the boundary estimators (described in the previous section) on data simulated by using Model 1. Then, we evaluate the detection of IBD/UPD regions on the artificial dataset of [35], in comparison with three well-known methods for LOH estimation. Using the same data, we also show the difference in the estimation when adjusting the parameters. Finally, we show the performance of gBPCR, when applied to real data.

With the current implementation, on a computer with dual CPU (AMD Opteron 250, 2.4 GHz) and 4 GB RAM, the algorithm needed almost two days to estimate the profile of an Affymetrix GeneChip Mapping 250K NspI sample (using *k*_*max *_= 50). Nevertheless, the computations can be performed by chromosome (and by arm for the longest chromosomes), reducing the computational cost. In any case, an optimized version of the code will be soon available.

### Comparison of the breakpoint estimators on simulated data

In Section "Methods", we have described several possible boundary estimators: ,  and . The last one actually defines a class of estimators which depend on the values of the thresholds *thr*_1 _and *thr*_2_. We tried several pairs of the following types of thresholds:

• "005" := *max*(0.005, quantile of **p **at 0.95)

• "01" := *max*(0.01, quantile of **p **at 0.95)

• "01 90" := *max*(0.01, quantile of **p **at 0.90)

• "mad" := *median*(**p**) + 3 * *mad*(**p**)

where *mad *is the median absolute deviation and **p **is the vector of posterior probabilities to be a breakpoint. All these thresholds derive from different definitions of which probability values are to be considered significant.

We assessed the quality of all the estimators of *k*_0 _and **t**^0 ^considered, by applying them on two artificial datasets (called datasets A and B), each of 100 samples. We used as prior probabilities of heterozygosity the frequencies of heterozygosity (in the CEU population of the HapMap project [29]) given by the annotation file of the Affymetrix GeneChip Mapping 250K NspI microarray. Just for illustrative purpose and because of limited computational time, we considered only the SNPs of a single chromosome (chromosome 22), hence the number of data points in each sample is *n *= 2520. Since our complete model (Model 3) does not provide a realistic way to simulate IBD/UPD regions and the identification of gained regions depends mainly on copy number data, the samples were generated using Model 1.

#### Simulation description

Since the Model 1 assumes to know the estimated copy number profile given by mBPCR, for both datasets, we fixed the estimated segment number  = 15, the estimated boundaries  = (0, 27, 31, 161, 273, 585, 633, 1006, 1050, 1054, 1309, 1607, 1754, 2100, 2432, 2520) (generated uniformly random given  = 15) and the prior distribution of **Z **(see Supplementary Table S.1 in Additional file 2, for dataset *A*, and Table 2, for dataset *B*). The profiles of the samples in dataset *A *should be estimated easily, since in each segment the prior distribution of *Z *is quite peaked.

**Table 2:** Prior distribution of Z in the simulated dataset B.

	segment														
	
prior	I	II	III	IV	V	VI	VII	VIII	IX	X	XI	XII	XIII	XIV	XV
P(^*Z*^*p *^= 2)^	0	0.1	0	0.1	0.5	0.1	0	0	0.1	0.5	0	0.1	0.5	0.1	0
P(^*Z*^*p *^= 0)^	0.1	0.6	0.1	0.6	0.4	0.6	0.1	0.1	0.6	0.4	0.1	0.6	0.4	0.6	0.1
P(^*Z*^*p *^= -1)^	0.6	0.3	0.6	0.3	0.1	0.3	0.6	0.4	0.3	0.1	0.6	0.3	0.1	0.3	0.6
P(^*Z*^*p *^= -2)^	0.3	0	0.3	0	0	0	0.3	0.5	0	0	0.3	0	0	0	0.3

Given the previous parameters (,  and the **Z **prior) and the estimated values of the other parameters of the model, we used the following steps to generate each LOH sample:

1. we generated a true profile of the homozygous status in normal cells **X**^*N*^, by using the prior probabilities of heterozygosity, described previously;

2. we generated a true copy number event profile , by using the prior distribution of **Z **(notice that in some cases the final profile can have less than 15 segments, since, if consecutive segments have the same copy number value, then they are joined together);

3. given the true copy number event profile and the profile of the homozygous status in normal cells, we generated **Y **(the profile of the homozygous status in cancer cells detected by the genotype calling algorithm), by using the conditional probability distributions of Model 1.

#### Results of the comparisons

To evaluate the performance of the estimators, we computed several error measures regarding the estimation of the number of segments (0-1, absolute and squared errors), the boundary estimation (binary error, sensitivity and false discovery rate, FDR) and the profile estimation (sum of squared distance, SSQ, sum 0-1 error, sensitivity and FDR for all copy number events). The explanation of these error measures can be found in Section S.5 in Additional file 2.

By applying the pairs of estimators (, ),(, ), and () to dataset *A*, the latter two appeared the best performing methods with respect to the error measures considered (see for example Table 3).

**Table 3:** Comparison among the breakpoint estimators with respect to error measures regarding copy number aberration detection.

dataset	method	sum 0-1 err	SSQ	
	(, )	51.53	86.08	0.19
*A*	(, )	146.91	596.78	0.49
	()	91.99	345.64	0.37

	(, )	421.79	1226.59	0.70
	()	110.39	287.21	0.34
	()	109.39	286.15	0.34
*B*	()	141.65	370.78	0.38
	()	154.56	424.2	0.41
	()	109.39	286.15	0.34
	()	111.75	283.77	0.34

The table shows some error measures regarding the copy number event estimation obtained with several methods on datasets *A *and *B*. While (, ) outperforms the other methods on dataset *A*, on *B *it obtains a poor estimation of the copy number events in comparison with the other methods. On dataset *B*, the methods which achieve the lowest errors are: (), (), () and ().

Based upon these results, we decided to not apply the estimators (, ) on dataset *B *and to try other paired thresholds for , in order to reduce the FDR of the boundary estimation. By looking globally at the results of all error measures (see Table 3, Supplementary Tables S.2-S.5 and Supplementary Figures S.2 and S.3 in Additional file 2), we can suggest the use of the following pairs of estimators: (), () or (). Moreover, from the study of the behavior of () and (), we can understand the role of the two thresholds in our algorithm for the determination of the maxima in a multimodal function (see Section S.4 in Additional file 2). The threshold *thr*_1 _is used to decide which points belong to the same peak: all the points, between two regions of points below *thr*_1_, are considered in the same peak. Hence, with a low threshold, more points are considered belonging to the same peak and thus we can eliminate lot of false breakpoints (like in ()). But, at the same time, if two true peaks are close, then it is possible that they are considered as only one peak, losing a true breakpoint (low sensitivity). Instead, the threshold *thr*_2 _is used to choose which estimated breakpoints are significant for the regression, i.e. if their posterior probabilities are to be considered different from zero. Therefore, using a lower value of *thr*_2_, we select a higher number of breakpoints obtaining a higher percentage of both false ones (high FDR) and true ones (high sensitivity, as in ()).

A detailed description of the results obtained in the comparison is in Section S.5 in Additional file 2.

### Comparisons on simulated data with LOH regions

In order to evaluate the IBD/UPD detection of gBPCR, we applied it to simulated data of [35]. These data are based on three real samples of the HapMap dataset (see [1]), obtained with the Affymetrix GeneChip Mapping 250K NspI. For each sample and signal to noise ratio (SNR) value, the authors simulated two profiles: one with regions of copy-neutral LOH and one with regions of loss. In both cases the number of regions was 50 and their width ranged from 20 SNPs to a whole chromosome. The values of SNR considered were 5, 2 and 1.25. Therefore, the total number of samples was 18, because for each normal sample we had two LOH profiles and three SNR values. The authors simulated the noise and the aberrations at probe level intensity saving the data in .CEL file format, thus we used BRLMM [26] to extract the genotyping data and CNAT 4.01 [36] for the raw copy number data.

Similar to [35], the estimation of gBPCR was compared with the ones given by three well-known methods in the field: dChip [16], CNAT 4.01 [36] and PennCNV [24]. The evaluation has been done by computing the true positive rate (TPR) and the false positive rate (FPR), i.e. the proportion of SNPs inside the LOH regions that are correctly identified (as belonging to a LOH region) and the proportion of SNPs outside these segments that are wrongly identified (as belonging to them), respectively. We used (), () or () as paired estimators of the number of segments and the boundaries, and either *p*_*upd *_= 10^-3 ^or *p*_*upd *_= 10^-4 ^mas the prior probability of IBD/UPD.

Since CNAT does not consider the *NoCall *SNPs (called **non-informative **SNPs) for the estimation of the LOH profile, we compared the TPR and FPR computed using only either the informative or the non-informative SNPs (see Supplementary Figures S.4, S.5, S.6 and S.7 in Additional file 2).

**Figure 6:**
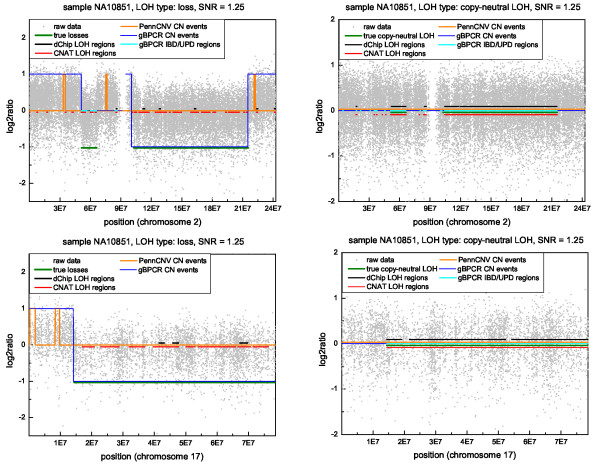
**Examples of profile estimation**. The plot shows four examples of chromosomic profile estimation in samples with SNR = 1.25 (high noise). The version of gBPCR employed was the one which uses () and *p*_*upd *_= 10^-4^. As notations: 1 corresponds to gain, 0 to normal status, -1 to loss. IBD/UPD regions and unspecified LOH regions are depicted with values close to zero. All methods (apart PennCNV) are able to identify the copy-neutral LOH regions, but sometimes dChip divides the biggest lesions in small ones. Only gBPCR and CNAT are able to detect LOH regions due to deletions and in this case CNAT divides the biggest aberrations into small regions of LOH.

Overall, all versions of gBPCR behaved similarly on these data and they outperformed PennCNV, CNAT and dChip. Moreover, dChip failed to give a good estimation in presence of high noise, while PennCNV did not detect almost any LOH aberration. Four examples of profile estimation in samples with SNR = 1.25 (high noise) are shown in Figure 6 (their corresponding LOH data are plotted in Supplementary Figure S.8 in Additional file 2). Regarding the copy-neutral LOH estimation, all methods (apart from PennCNV) were able to identify the aberrations, but sometimes dChip divided the biggest lesions into small regions of aberration (e.g. the plot at the bottom right-hand side of Figure 6). Instead, only gBPCR and CNAT were usually able to detect LOH regions due to deletions. In this case, CNAT divided the biggest aberrations in small regions of LOH, losing part of the lesions. In Figure 6, we can also appreciate the differences in the estimation of the regions of gain between gBPCR and PennCNV. In both examples with regions of loss (the plots at the top and at the bottom left-hand side), the segments outside the losses represent gains. gBPCR failed to identify only one of these lesions, instead PennCNV did not recognize almost any of them (for thoroughness, we plotted also the copy number events, estimated by the HMM methods implemented in dChip and CNAT, in Supplementary Figure S.9 in Additional file 2). In the next section, by applying gBPCR to a real dataset from [23], we will be able to discuss its performance in the identification of genomic gains, depending on the copy number of the alleles (e.g. *CN *= 4 and both alleles have *CN *= 2 or one allele has *CN *= 1 and the other *CN *= 3).

Finally, we also evaluated the effect of the adjustment of the model parameters related to the *NoCall *detection (see Section "Methods"), using the same data. At low or medium noise, no significant differences in the goodness of the estimation could be observed (see, for example, Supplementary Figure S.10 in Additional file 2). Instead, in presence of high noise, the FPR regarding the IBD/UPD detection without the adjustment of the model parameters was close to one. In fact, in this situation a segment with normal copy number is more often classified as IBD/UPD, since the *NoCall *rate is higher and, without the correction, the IBD/UPD segments are allowed to contain a higher percentage of *NoCall*s with respect to the normal ones. Instead, with the adjustment, all types of regions are allowed to have a higher number of *NoCall*s in proportion to the noise, obtaining a less biased estimation.

In conclusion, we suggest to use () or () with *p*_*upd *_= 10^-4^, due to their good results obtained on the non-informative SNPs. A detailed description of all the results is in Section S.6 in Additional file 2.

### Application to real data

In this subsection, we show the behavior of gBPCR on three real datasets. The first dataset consisted of eight paired cancer samples of patients affected by chronic lymphocytic leukemia (CLL), which then developed in diffuse large B-cell lymphoma (DLBCL), see [37,38]. For two patients we had also a third sample, thus the total number of samples was 18. The second dataset consisted of 18 patients affected by CLL, see [39]. For both of these datasets, genome-wide DNA profiles were obtained using the GeneChip Human Mapping 250K NspI (Affymetrix, Santa Clara, CA, USA). The genotype calls were calculated with BRLMM [26] using 46 Caucasian normal female samples of the HapMap Project as reference samples and the raw copy number data were retrieved using CNAT 4.01 [36]. In [37-39], the copy number of some genomic regions was also measured with fluorescent *in situ *hybridization (FISH). Therefore, on these regions we compared the copy number event estimated by gBPCR with the copy number measured by FISH. Moreover, since samples coming from the same patient should present the same copy-neutral LOH regions (the germ line ones) for the majority of the genome, we used the two patients with three samples to evaluate the IBD/UPD detection of gBPCR.

The third dataset was a dilution series of the CRL-2324 breast cancer cell line from [23]. The series comprised 12 samples, corresponding to the following percentages of tumor content: 0, 10, 14, 21, 23, 30, 34, 45, 47, 50, 79, 100. The genome-wide DNA profiles were obtained using Illumina 370K BeadChips. The authors preprocessed the data with BeadStudio software (Illumina Inc.) and we used both the genotyping and logRratio data available at [40]. In [23], the authors chose eight genomic aberrations and compared the estimation given by their method (called BAFsegmentation) with the ones of the following algorithms: dChip [16], PennCNV [24], QuantiSNP [20] and SOMATICs [18]. Thus, we compared the estimations of these genomic regions given by gBPCR with the ones given by the previous methods. We also used these data to evaluate the performance of gBPCR in the detection of gains, for different values of the allelic copy numbers.

Based on the previous results on simulated data, for the analysis of these real data, we used: (), as paired estimators of the number of segments and the boundaries, and *p*_*upd *_= 10^-4 ^as prior probability of IBD/UPD.

#### Results regarding the identification of the copy number changes in CLL samples

**Figure 7:**
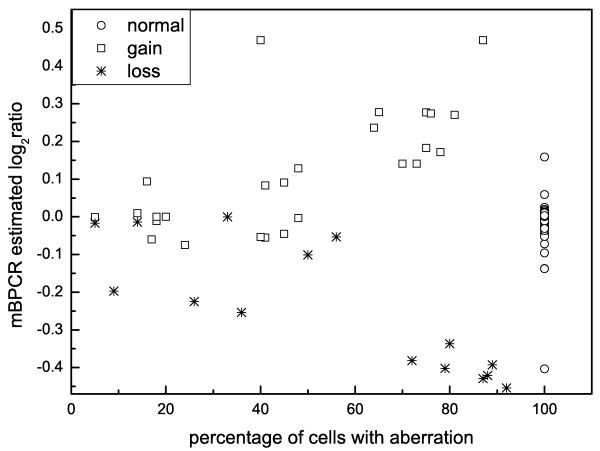
**Example of copy number event classification**. The plot shows the estimated log_2_ratio values (given by mBPCR), as function of the estimated percentage of cells bearing the aberration (given by FISH). The aberrations considered in the graph were identified by FISH on 18 patients of [37,38], for a total of 133 interrogated genomic regions. The copy number changes are classified as loss, gain or normal, using the results given by the FISH. For the normal regions, we set the percentage as 100%. The estimated log_2_ratio is the one of the genomic region interrogated by FISH. We can observe that only the aberrations borne in at least 60% of the cells are clearly separated.

We recall that an individual cancer sample can represent a mixture of neoplastic and normal cells. Moreover, the tumor cells themselves do not represent a genetically homogeneous population, since individual genetic lesion might be present in only a fraction of the cells. In fact, Figure 7 shows that the log_2_ratio values corresponding to normal, gain, loss regions are sufficiently well separated only when the copy number changes are borne in at least 60% of the cells of the DNA sample. As a consequence, we aim to detect the copy number changes borne in at least the 60% of the cells, otherwise we cannot ensure that the identified aberrations are true and not due to the noise of the microarray data (the noise is so high that aberrations borne in only a small percentage of cells can be seen as noise and viceversa). To detect aberrations in even less cell content, it is sufficient to change the prior of **Z **with thresholds closer to zero. In practice, the prior of **Z **influences more the discovery of the gains than the one of the other copy number events, because the determination of gains depends mainly on the estimated log_2_ratio values (rather than the LOH data).

In the samples considered for the comparison, we had a total of 169 regions measured by FISH (which provides also an estimate of the percentage of cells bearing the aberration): 38 regions were gains or losses in at least 60% of the cells (called **detectable aberrations**), 33 were gains or losses in less than 60% (called **less detectable aberrations**) and 98 were identified as normal segments. Regarding the detectable aberrations, only two copy number events were not identified by our algorithm. One loss was not found, because the estimated log_2_ratio was very close to zero, and the other, because of a different percentage of *Het *SNPs from what was expected by our algorithm. We discovered 13 of the 33 less detectable copy number changes and we also detected two of the 98 normal segments as aberrations, but one of these copy number changes was equal to the one discovered in the same region of the paired sample, thus it was likely to be true.

Instead, by simply using the thresholds of the prior of *Z *on the profiles estimated by mBPCR for the classification of the copy number events (similarly to what is usually done), we detected one alteration less than what found by gBPCR and other 5 normal regions were seen as aberrations.

For the analysis of the results, we have to consider that the samples used for FISH came from peripheral blood, for the CLL samples, and from paraffn embedded tissues or lymph node, for the DLBCLs. Because of the consequently different cell content, in the former case, the results are better estimated. Moreover, the samples used for microarray and FISH might not be exactly the same, hence the percentage of cells which carry the aberrations can be different and a discordance between the two techniques is possible. Thus, gBPCR performed well in estimating the copy number changes on these samples.

#### Results regarding IBD/UPD region detection in CLL samples

For the evaluation of the IBD/UPD region detection, we considered the two patients with three samples. For the first patient (called Patient 1), we had: one matched normal DNA sample extracted from peripheral blood granulocytes (called Sample 1.1), one sample from neoplastic cells at CLL phase (called Sample 1.2) and the last one from neoplastic cells at DLBCL phase (called Sample 1.3). For the second patient (Patient 2), we had: one sample from neoplastic cells at CLL phase (called Sample 2.1), one at DLBCL phase (called Sample 2.2) and the last one from neoplastic cells at a further progression of the DLBCL (called Sample 2.3).

Applying gBPCR to the three samples of Patient 1, we found that the number of aberrations in each sample increased with the progression of the disease. The lower number of segments discovered in Sample 1.1 could also be due to a higher *NoCall *rate in comparison to the other samples. The same happened for Sample 2.3 of Patient 2.

We compared the IBD/UPD segments found in the three samples of each patient and we divided them into three classes (see Supplementary Table S.6 in Additional file 2):

• equal regions: segments that are exactly the same in two or three samples;

• overlapping regions: segments that are common in at least two samples but do not have the same boundaries;

• single sample regions: the remaining segments.

Then, we defined the number of distinct regions as the sum of all these regions and the number of validated ones as the sum of all types of regions except the single sample regions. The proportions of equal and overlapping regions were similar in the two patients and the validated regions were 73% of the distinct regions detected in Patient 1 and 79% of the distinct regions in Patient 2. The single sample regions were about the 21% of the distinct regions in Patient 2, but the majority of them had length less than 50 SNPs. Instead, since the samples of Patient 1 belonged to different stages of the disease, in this patient we found a higher number of single sample regions and most of them were wider than 50 SNPs. In fact, the majority of these regions was detected in Sample 1.3, thus they were likely to be somatic.

#### Results regarding the identification of genomic aberrations in the dilution series

In [23], the authors observed that the BeadStudio normalization produced copy number profiles which were centered differently as the tumor content decreased and, as a consequence, many algorithms wrongly assigned the type of genetic aberration. Therefore, they evaluated the methods by considering only if they found any aberration in the eight regions considered, without looking at the type of aberration.

Due to this variation in centering the normal copy number, we estimated the histogram of the estimated log_2_ratio values (which is used for the definition of the prior of **Z**), separately by using only samples with similar tumor content. Nevertheless, this shrewdness was not suffcient to well distinguish the peaks of the histogram in some cases.

For all samples, we computed the sensitivity in detecting the eight aberrations considered in [23]. For each of them, the calculations were done in two ways: by looking if gBPCR found any aberration (like in [23]) and by looking if it found the correct lesion (see Supplementary Figures S.11 and S.12 in Additional file 2). By comparing the results obtained by gBPCR using the first type of sensitivity with the ones given by the other methods in Figure 7 of [23], we can observe that gBPCR outperformed dChip and PennCNV and often also QuantiSNP. Sometimes it also performed better than BAFsegmentation and SOMATICs in the detection of the regions of gain. Moreover, occasionally gBPCR had a non-zero sensitivity in the normal sample, because it detected small IBD/UPD regions. By looking at the results obtained by gBPCR with the second type of sensitivity, we can notice that the correct aberration was usually detected in samples with at least 60% of tumor content and the sensitivity was still often higher than the one of dChip and PennCNV.

Finally, we computed the sensitivity of gBPCR for eight regions of gain, to evaluate its performance depending on the value of the copy number of the alleles. For all eight lesions, the total copy number was four. Instead, the minor allele copy number (*maCN *, i.e. the copy number of the allele less frequent in a normal population) changed from two to zero. The selection of these regions of gain was based on the estimated genomic profile of CRL-2324, provided by The Cancer Genome Project at the Wellcome Trust Sanger Institute and available at [41]. For each aberration, the sensitivity was computed in two ways: by looking if the region was identified as a gain and by looking if it was detected as either a gain or an IBD/UPD segment (see Supplementary Figure S.13 in Additional file 2).

The differences between the two types of sensitivity were observed for some percentages of tumor content, in gains with *maCN *= 2 or *maCN *= 0. Regarding the lesions with *maCN *= 2, a small part of the gain was identified as IBD/UPD region in few cases with a small percentage of tumor content. This phenomenon was due to the presence of a high percentage of homozygous SNPs with copy number close to the normal copy number. For the same reason, the whole gain 6q22.31 (*maCN *= 0) was identified as an IBD/UPD region at 100% and 79% percentages of tumor content and the same happened also for a part of 6q15 (*maCN *= 0) at 79%. As we explained in Section "Method", the detection of the gains highly depends on the copy number value. Thus, if the copy number of a region of gain is close to the normal value, it is identified as either normal or IBD/UPD, depending on the homozygous status of the SNPs inside it. Therefore, the performance of gBPCR depends mainly on the quality of the copy number data and not on the value of the copy number of the alleles.

## Conclusions

We have derived a new algorithm (called gBPCR) for the simultaneous estimation of copy number changes and IBD/UPD regions, by using both copy number and genotyping data. To the best of our knowledge, only one other algorithm exists which uses the same input data for the same purpose [17], but it does not appear appropriate for data coming from a DNA sample of a mixture of cell populations (like cancer DNA samples).

Our model takes into account the errors due to both the microarray procedure and the biological processes that lead to aberrations affecting the DNA copy number and the homozygous status. Because of the complexity of the algorithm and the high noise of the real data, we introduced new estimators to improve the detection of the breakpoints. On the basis of the results on simulated data, we selected the best performing one: ().

On the artificial dataset of [35] (and especially in samples with high noise), gBPCR outperformed three well-known methods which estimate regions of LOH: dChip [16], CNAT [36] and PennCNV [24]. We also tested gBPCR on real data. On 36 CLL samples [37-39], we found a high agreement between the copy number changes estimated by gBPCR and the ones obtained by FISH (used as reference). Moreover, on two patients with three samples we could validate at least 73% of the identified IBD/UPD segments. On the samples of the CRL-2324 dilution series of [23], we showed that in samples with at least 60% of tumor content, gBPCR was able to detect the genomic aberrations, while with less tumor content only some aberrations could be seen. Moreover, on these data gBPCR outperformed dChip [16] and PennCNV [24] and sometimes QuantiSNP [20]. Since other methods (SOMATICs [18] and BAFsegmentation [23]), which use the allelic copy number information, seemed to perform well, as future work we intend to add also this useful information in our model.

## Availability and requirements

**Project name**: gBPCR.

**Project home page**: http://www.idsia.ch/~paola/gBPCR/.

**Operating systems**: the software should run in Linux, Mac-OS or Windows. Tests were performed on Windows and Linux systems.

**Programming language**: R.

**Other requirements**: none.

**Licence**: GNU GPL.

**Any restrictions to use by non-academics**: none.

## Authors' contributions

PMVR carried out the study and wrote the manuscript. MH and IK supervised the statistical analysis. FB supervised the validation study and provided the real data. All authors read and approved the final manuscript.

## Supplementary Material

Additional file 1 **gBPCR source code**. This zipped file contains the source code of the gBPCR algorithm in R, including help files, sample data and examples. Click here for file

Additional file 2 **Supplementary material**. This file contains: 1) the description of the estimation of the parameters of the likelihood, 2) the explanation of the estimation of density of the estimated log2ratio levels, 3) explicit formulae of some quantities employed in the dynamic programming used to implement our method, 4) the explanation of an algorithm for the determination of the maxima of a multimodal function, 5) detailed description of the results obtained on simulated data, 6) some supplementary tables and 7) some supplementary figures. Click here for file
