# Supplementary material for: An integrated Bayesian analysis of LOH and copy number data
Source: BMC Bioinformatics. 2010 Jun 15;11:321. doi: 10.1186/1471-2105-11-321 (PMC2912301; doi:10.1186/1471-2105-11-321)
Supplement: Additional file 1 — gBPCR source code. This zipped file contains the source code of the gBPCR algorithm in R, including help files, sample data and examples. [file 1471-2105-11-321-S1.ZIP › gBPCRsource_code/html/writeEstProfileGBPCR.html]

R: Write the estimated profile of genomic aberrations

|  |  |
| --- | --- |
| writeEstProfileGBPCR {gBPCR} | R Documentation |

## Write the estimated profile of genomic aberrations

### Description

Function to write nicely the results of estimation of the genomic aberrations. The function either writes the tables
directly on a tab delimited file or returns the corresponding tables.

### Usage

```
  writeEstProfileGBPCR(path='', sampleName='', snpName, chr, position, rawLogratio, estLogratio,
                  chrToBeWritten, estState, estBoundaries=NULL, postProbT=NULL)
```

### Arguments

|  |  |
| --- | --- |
| `path` | path of the folder where the user wants to write the results of the estimation (it must end with '\' in windows, or '//' in linux). If `path=''`, they will be written in the working directory. If `path=NULL`, the tables will not be written on a file, but only returned by the function. |
| `sampleName` | name of the sample. If the name of the sample if provided, it is used to named the files. |
| `snpName` | array containing the name of each probe |
| `chr` | array containing the name of the chromosome to which each probe belongs. The possible values of the elements of `chr` are: the integers from 1 to 22, 'X' and 'Y'. |
| `position` | array containing the physical position of each probe |
| `rawLogratio` | array containing the log2ratio of the raw copy number data |
| `estLogratio` | array containing the estimated log2ratio of the copy number as a piecewise constant function (preferably by using mBPCR) |
| `chrToBeWritten` | array containing the name of the estimated chromosomes, of which the user wants to write the results. The possible values of the chromosomes are: the integers from 1 to 22, 'X' and 'Y'. |
| `estGenAber` | array containing the estimated genomic aberrations as a piecewise constant function by using gBPCR (it has the same length of `position` and `chr`). The aberrations are codified as following: `A` (high amplification), `G` (gain), `N` (normal state), `L` (loss of one copy), `HD` (homozygous deletion, i.e. loss of two copies), `IBD/UPD` (copy-neutral LOH). |
| `estBoundaries` | list containing the vectors of the estimated breakpoints, for each of the chromosomes mentioned in `chrToBeWritten`. If `estBoundaries=NULL`, then this information is not written. |
| `postProbT` | list containing the vectors of the posterior probabilities to be a breakpoint of the estimated breakpoints, for each of the chromosomes mentioned in `chrToBeWritten`. If `postProbT=NULL`, then this information is not written in the file containing the estimated breakpoints. |

### Details

The function writes returns at maximum two tables:

-one containing the estimated profile with gBPCR in terms of `estGenAber` (the columns are: 'SNPname', 'chromosome', 'position', 'rawLog2ratio', 'mBPCRestimate', 'gBPCRestimate')

-one containing a summary about the estimated profile with gBPCR in terms of `estGenAber` (the columns are: 'SNPname(start)', 'SNPname(end)', 'chromosome', 'position(start)', 'position(end)', 'nProbes', 'gBPCRestimate' and, eventually, 'breakpointPostProb'). This table is not created if `estBoundaries=NULL`.

### Examples

```
###Before using the following commands, set "gBPCR" as working directory

###import the 250K nsp data of sample NA10851_LOH_20
path <- paste(getwd(), "/data/NA10851_LOH_20.dat",sep='')
sample <- importGenomicData(path, NRowSkip=1)
###we select only the data belonging to the first part of chromosome 7
chr1 <- sample$chr
chr1[chr1==7][-(1:500)] <- 8
pHetData <- xPrior(typeArray='Affy250Knsp', race='CEU')
pHet1 <- array(dim=length(chr1))
pHet1[chr1==7] <- pHetData$pHet[pHetData$chrPHet == 7][1:500] 
load(paste(getwd(),'/data/paramHist20.RData',sep=''))
thrHist <- createThr(paramHist)
###estimation of the profile of the part of interest of chromosome 7
results <- estProfileWithGBPCR(snpName=sample$snpName, chr=chr1, position=sample$position, call=sample$call, rawLogratio=sample$rawLogratio, estLogratio=sample$estLogratio, thrHist=thrHist, chrToBeAnalyzed=7, maxProbeNumber=1000, pHet=pHet1, kMax=10)
###write the estimated profile in a nice format
results2 <- writeEstProfileGBPCR(path=NULL, sampleName='NA10851_LOH_20', snpName=sample$snpName, chr=chr1, position=sample$position, rawLogratio=sample$rawLogratio, estLogratio=sample$estLogratio, chrToBeWritten=7, estGenAber=results$estGenAber, estBoundaries=results$estBoundaries, postProbT=results$postProbT) 
results2$gBPCRbreakpoints
```

---

[Package Index]
