# Supplementary material for: An integrated Bayesian analysis of LOH and copy number data
Source: BMC Bioinformatics. 2010 Jun 15;11:321. doi: 10.1186/1471-2105-11-321 (PMC2912301; doi:10.1186/1471-2105-11-321)
Supplement: Additional file 1 — gBPCR source code. This zipped file contains the source code of the gBPCR algorithm in R, including help files, sample data and examples. [file 1471-2105-11-321-S1.ZIP › gBPCRsource_code/html/00Index.html]

R: gBPCR

# gBPCR: Bayesian Piecewise Constant Regression for LOH and CN data

---

## Documentation for package `gBPCR' version 1.0


## Help Pages

|  |  |
| --- | --- |
| cna2logCn | Copy number aberration conversion to log2ratio symbols |
| codification | Codification of genotyping data |
| createThr | Estimate the thresholds of the copy number aberrations |
| estGlobParam | Estimate global parameters of copy number data |
| estProfileWithGBPCR | Estimate the profile of genomic aberrations of some chromosomes of a sample |
| genAber2state | Genomic aberration conversion from abbreviations to state values |
| importCNData | Import genomic data |
| logAdd | Overflow-safe computation of the logarithm of a sum |
| logCn2cna | Copy number aberration (CNA) conversion from log2ratio symbols to CNA abbreviations |
| plotEstProfileGBPCR | Plot the estimated profile of genomic aberrations |
| state2genAber | Genomic aberration conversion from state values to abbreviations |
| stateConversion | Genomic aberration conversion from state values to CNAs and copy-neutral LOH regions |
| writeEstProfileGBPCR | Write the estimated profile of genomic aberrations |
| xPrior | Import the probabilities of heterozygosity |
| gBPCR-internal | Internal gBPCR functions |
