# Supplementary material for: An integrated Bayesian analysis of LOH and copy number data
Source: BMC Bioinformatics. 2010 Jun 15;11:321. doi: 10.1186/1471-2105-11-321 (PMC2912301; doi:10.1186/1471-2105-11-321)
Supplement: Additional file 1 — gBPCR source code. This zipped file contains the source code of the gBPCR algorithm in R, including help files, sample data and examples. [file 1471-2105-11-321-S1.ZIP › gBPCRsource_code/html/cna2logCn.html]

R: Copy number aberration conversion to log2ratio symbols

|  |  |
| --- | --- |
| cna2logCn {gBPCR} | R Documentation |

## Copy number aberration conversion to log2ratio symbols

### Description

Function that converts the copy number aberrations in numbers corresponding to their "symbolic" log2ratio values.

### Usage

```
  cna2logCn(cna)
```

### Arguments

|  |  |
| --- | --- |
| `cna` | array containing the copy number aberrations (CNAs). The CNAs are codified as following: `A` (high amplification), `G` (gain), `N` (normal copy number), `L` (loss of one copy), `HD` (homozygous deletion, i.e. loss of two copies). |

### Value

A numeric array with elements equal to: `2.5` at `A` (high amplification), `1` at `G` (gain), `0` at `N` (normal copy number),
`-1` at `L` (loss of one copy), `-2.5` at `HD` (homozygous deletion, i.e. loss of two copies).

### Note

The inverse function is called `logCn2cna`.

### See Also

`logCn2cna`,`stateConversion`

### Examples

```
##let us define an array of copy number aberrations  
cna <- c(array("A", dim=200), array("N", dim=50), array("L", dim=100), array("HD", dim=300))
##now we convert the copy number aberrations by using cna2logCn and we plot them
plot(cna2logCn(cna))
```

---

[Package Index]
