# Supplementary material for: An integrated Bayesian analysis of LOH and copy number data
Source: BMC Bioinformatics. 2010 Jun 15;11:321. doi: 10.1186/1471-2105-11-321 (PMC2912301; doi:10.1186/1471-2105-11-321)
Supplement: Additional file 1 — gBPCR source code. This zipped file contains the source code of the gBPCR algorithm in R, including help files, sample data and examples. [file 1471-2105-11-321-S1.ZIP › gBPCRsource_code/html/codification.html]

R: Codification of genotyping data

|  |  |
| --- | --- |
| codification {gBPCR} | R Documentation |

## Codification of genotyping data

### Description

Function that codifies the genotyping data in numbers (it is used in `estProfileWithGBPCR` for computational purpose).

### Usage

```
  codification(call, callName=NULL)
```

### Arguments

|  |  |
| --- | --- |
| `call` | array containing the genotyping data |
| `callName` | list containing four fiels corresponding to the symbols used in the vector `call` to codify the genotype. Namely, `callA` contains the symbol of the genotype AA, `callB` of the genotype BB, `callAB` of the genotype AB and `callNC` of the NoCall. If `callName` is not specified, then `callA = 'AA'`, `callB = 'BB'`, `callAB = 'AB'` and `callNC = 'NC'` |

### Value

A numeric array with elements equal to: `1` at the heterozygous SNPs (i.e. AB genotype), `0` at the homozygous SNPs (i.e. AA or BB genotype)
and `-1` at the NoCall SNPs.

### See Also

`estProfileWithGBPCR`,

### Examples

```
##let us define an array of genotyping data  
call <- c(array("A", dim=200), array("B", dim=50), array("NC", dim=100), array("AB", dim=300))
##we define the callName object compatible with our genotyping data
ourCallName <- list(
            callA  = 'A',
            callB  = 'B',
            callAB = 'AB',
            callNC = 'NC')
##now we codify the genotyping data by using codification and we plot them
plot(codification(call, callName=ourCallName))
```

---

[Package Index]
