# Supplementary material for: An integrated Bayesian analysis of LOH and copy number data
Source: BMC Bioinformatics. 2010 Jun 15;11:321. doi: 10.1186/1471-2105-11-321 (PMC2912301; doi:10.1186/1471-2105-11-321)
Supplement: Additional file 1 — gBPCR source code. This zipped file contains the source code of the gBPCR algorithm in R, including help files, sample data and examples. [file 1471-2105-11-321-S1.ZIP › gBPCRsource_code/html/estGlobParam.html]

R: Estimate global parameters of copy number data

|  |  |
| --- | --- |
| estGlobParam {mBPCR} | R Documentation |

## Estimate global parameters of copy number data

### Description

Function to estimate the global parameters of copy number data (in mBPCR): the mean and the variance of the segment levels (called `nu` and `rhoSquare`, respectively), the variance of the noise (`sigmaSquare`). It is possible
to choose the estimator of `rhoSquare` (i.e. either *hat{rho}\_1^2* or *hat{rho}^2*) and by default *hat{rho}\_1^2* is used.

### Usage

```
  estGlobParam(y, nu=NULL, rhoSquare=NULL, sigmaSquare=NULL, typeEstRho=1)
```

### Arguments

|  |  |
| --- | --- |
| `y` | array containing the log2ratio of the copy number data |
| `nu` | mean of the segment levels. If `nu=NULL`, then the algorithm estimates it on the sample. |
| `rhoSquare` | variance of the segment levels. If `rhoSquare=NULL`, then the algorithm estimates it on the sample. |
| `sigmaSquare` | variance of the noise. If `sigmaSquare=NULL`, then the algorithm estimates it on the sample. |
| `typeEstRho` | choice of the estimator of `rhoSquare`. If `typeEstRho=1`, then the algorithm estimates `rhoSquare` with *hat{rho}\_1^2*, while if `typeEstRho=0`, it estimates `rhoSquare` with *hat{rho}^2*. |

### Value

A list containing:

|  |  |
| --- | --- |
| `nu` |  |
| `rhoSquare` |  |
| `sigmaSquare` |  |

### References

Rancoita, P. M. V., Hutter, M., Bertoni, F., Kwee, I. (2009).
Bayesian DNA copy number analysis. *BMC Bioinformatics* 10: 10.
  
http://www.idsia.ch/~paola/mBPCR

### Examples

```
###Before using the following commands, set "gBPCR" as working directory

###import the 250K nsp data of sample NA10851_LOH_20
path <- paste(getwd(), "/data/NA10851_LOH_20.dat",sep='')
sample <- importGenomicData(path, NRowSkip=1)
###estimation of all the global parameters (the variance of the segment is estimated with \eqn{\hat{\rho}^2_1})
estGlobParam(sample$rawLogratio)
```

---

[Package Index]
