# Supplementary material for: An integrated Bayesian analysis of LOH and copy number data
Source: BMC Bioinformatics. 2010 Jun 15;11:321. doi: 10.1186/1471-2105-11-321 (PMC2912301; doi:10.1186/1471-2105-11-321)
Supplement: Additional file 1 — gBPCR source code. This zipped file contains the source code of the gBPCR algorithm in R, including help files, sample data and examples. [file 1471-2105-11-321-S1.ZIP › gBPCRsource_code/html/estProfileWithGBPCR.html]

R: Estimate the profile of genomic aberrations of some chromosomes of a sample

|  |  |
| --- | --- |
| estProfileWithGBPCR {gBPCR} | R Documentation |

## Estimate the profile of genomic aberrations of some chromosomes of a sample

### Description

Function to estimate the profile of genomic aberrations with a piecewise constant function using gBPCR. It is possible
to choose which estimator *hat{T}\_{Peak,thr\_1,thr\_2}* is used in the regression (i.e. either *hat{T}\_{Peak,01,01}*
or *hat{T}\_{Peak,mad,01}* or *hat{T}\_{Peak,01,mad}*) and by default *hat{T}\_{Peak,01,01}* is used.

### Usage

```
  estProfileWithGBPCR(snpName, chr, position, call, rawLogratio, estLogratio, thrHist, chrToBeAnalyzed,
                      maxProbeNumber, callName=NULL, pHet=NULL, typeArray='Affy250Knsp', race='CEU',
                      kMax=50, EstRho="NO", thr1="01", thr2="01", pUPD=0.0001, hg=17)
```

### Arguments

|  |  |
| --- | --- |
| `snpName` | array containing the name of each probe |
| `chr` | array containing the name of the chromosome to which each of the probes belongs. The possible values of the elements of `chr` are: the integers from 1 to 22, 'X' and 'Y'. |
| `position` | array containing the physical position of each probe |
| `call` | array containing the genotyping data |
| `rawLogratio` | array containing the log2ratio of the raw copy number data |
| `estLogratio` | array containing the estimated log2ratio of the copy number as a piecewise constant function (preferably by using mBPCR) |
| `thrHist` | array containing the thresholds for the classificaion of the log2ratio values in the copy number aberrations. The classification is done in the following way: `log2ratio ≤ thr[1]` : homozygous deletion, `thr[1] < log2ratio ≤ thr[2]` : loss, `thr[2] < log2ratio ≤ thr[3]` : normal, `thr[3] < log2ratio ≤ thr[4]` : gain, `log2ratio > thr[4]` : high amplification. |
| `chrToBeAnalyzed` | array containing the name of the chromosomes that the user wants to analyze. The possible values of the chromosomes are: the integers from 1 to 22, 'X' and 'Y'. |
| `maxProbeNumber` | maximum number of probes that a chromosome (or arm of a chromosome) can have to be analyzed. The procedure of profile estimation needs the computation of an array of length *(length(chromosome)+1)\*(length(chromosome)+2)/2*. To be sure to have set this parameter correctly, try to create the array `A <- array(1, dim=(maxProbeNumber+1)*(maxProbeNumber+2)/2)`, before starting with the estimation procedure. |
| `callName` | list containing four fiels corresponding to the symbols used in the vector `call` to codify the genotype. Namely, `callA` contains the symbol of the genotype AA, `callB` of the genotype BB, `callAB` of the genotype AB and `callNC` of the NoCall. If `callName` is not specified, then `callA = 'AA'`, `callB = 'BB'`, `callAB = 'AB'` and `callNC = 'NC'` |
| `pHet` | array containing the probability of heterozygosity of each SNP. If `pHet` is not specified, these probabilities will be retrieved in some annotation files by using the parameters `typeArray` and `race` |
| `typeArray` | denotes the type of microarray used (currently only the value `Affy250Knsp` is supported). The parameter is only used to retrieved the probabilities of heterozygosity, in case `pHet` is not specified |
| `race` | denotes the race of the patient. Currently the following values are supported: `CEU` (Utah residents with Northern and Western European ancestry from the CEPH collection), `CHB` (Han Chinese in Beijing, China), `JPT` (Japanese in Tokyo, Japan), `YRI` (Yoruban in Ibadan, Nigeria). The parameter is only used to retrieved the probabilities of heterozygosity, in case `pHet` is not specified |
| `kMax` | maximum number of segments |
| `EstRho` | the parameter denotes if and how estimate the variance of the log2ratio levels (`rhoSquare`, see package mBPCR). If `EstRho="NO"`, a default value is used, if `EstRho="YES"`, then the algorithm estimates `rhoSquare` with *hat{rho}\_1^2*, otherwise `EstRho` must contained the value of `rhoSquare` estimated by the user |
| `thr1` | choice of the first threshold used to compute the estimator *hat{T}\_{Peak,thr\_1,thr\_2}*. The following values are supported: "01", "mad". |
| `thr2` | choice of the second threshold used to compute the estimator *hat{T}\_{Peak,thr\_1,thr\_2}*. The following values are supported: "01", "mad". |
| `pUPD` | probability of occurrance of an IBD/UPD event. The default value is 0.0001. |
| `hg` | reference of human genome used for the annotation of the SNPs. The following value are supported: 17 and 18. |

### Details

By default, the function estimates the profile of genomic aberrations using gBPCR with *hat{T}\_{Peak,01,01}* as breakpoint estimator. It is
also possible to use either *hat{T}\_{Peak,mad,01}* or *hat{T}\_{Peak,01,mad}* as estimator of the breakpoints, by setting
`thr1='mad'` and `thr2='01'` or `thr1='01'` and `thr2='mad'`, respectively.

See function `writeEstProfileGBPCR`, to have the results in nicer tables or to write them on files.

See function `codification`, to have an example of how create the object `callName`.

### Value

A list containing:

|  |  |
| --- | --- |
| `estGenAber` | array containing the estimated genomic aberrations as a piecewise constant function by using gBPCR (it has the same length of `position` and `chr`). The aberrations are codified as following: `A` (high amplification), `G` (gain), `N` (normal state), `L` (loss of one copy), `HD` (homozygous deletion, i.e. loss of two copies), `IBD/UPD` (copy-neutral LOH). |
| `estCNA` | array containing the estimated copy number aberrations (CNAs) by using gBPCR (it has the same length of `position` and `chr`). The CNAs are codified as following: `A` (high amplification), `G` (gain), `N` (normal copy number), `L` (loss of one copy), `HD` (homozygous deletion, i.e. loss of two copies). |
| `estUPD` | array containing the estimated regions of IBD/UPD by using gBPCR (it has the same length of `position` and `chr`). The elements of the array are: `1`, if there is a copy-neutral LOH in the position, and `0`, otherwise. |
| `estBoundaries` | the list of estimated breakpoints for each of the analyzed chomosomes |
| `postProbT` | the list of the posterior probablity to be a breakpoint for each estimated breakpoint of the analyzed chomosomes |

`estGenAber`, `estCNA` and `estUPD` have the same length of `position`, hence their components,
corresponding to the not analyzed chromosomes, are equal to `NA`.

### References

Rancoita, P. M. V., Hutter, M., Bertoni, F., Kwee, I. (2010). An integrated Bayesian analysis of LOH and copy number data. Submitted.
  
http://www.idsia.ch/~paola/gBPCR

### See Also

`plotEstProfileGBPCR`, `writeEstProfileGBPCR`, `codification`, `xPrior`,`createThr`

### Examples

```
###Before using the following commands, set "gBPCR" as working directory

#EXAMPLE1
###import the 250K nsp data of sample NA10851_LOH_20
path <- paste(getwd(), "/data/NA10851_LOH_20.dat",sep='')
sample <- importGenomicData(path, NRowSkip=1)
###we select only the data belonging to the first part of chromosome 7
chr1 <- sample$chr
chr1[chr1==7][-(1:500)] <- 8
pHetData <- xPrior(typeArray='Affy250Knsp', race='CEU')
pHet1 <- array(dim=length(chr1))
pHet1[chr1==7] <- pHetData$pHet[pHetData$chrPHet == 7][1:500] 
load(paste(getwd(),'/data/paramHist20.RData',sep=''))
thrHist <- createThr(paramHist)
###estimation of the profile of the part of interest of chromosome 7
results <- estProfileWithGBPCR(snpName=sample$snpName, chr=chr1, position=sample$position, call=sample$call, rawLogratio=sample$rawLogratio, estLogratio=sample$estLogratio, thrHist=thrHist, chrToBeAnalyzed=7, maxProbeNumber=1000, pHet=pHet1, kMax=10)
###plot the estimated profile
UPD <- results$estUPD
UPD[UPD == 0] <- NA
UPD[UPD == 1] <- 0
plot(sample$rawLogratio[chr1 == 7])
points(sample$estLogratio[chr1 == 7],col=3,type='l',lwd=3)
points(cna2logCn(results$estCNA[chr1 == 7]),col=2,type='l',lwd=2)
points(UPD[chr1 == 7],col=5,type='l',lwd=2)
legend(x="bottomleft",legend=c('mBPCR','CNAs','IBD/UPD'),lty=c(1,1,1),col=c(3,2,5))

#EXAMPLE 2
###for the estimation of the profile of the whole chromosome 7, it is sufficient to use chr=sample$chr,
### the default parameters for pHet and kMax and augment maxProbeNumber
###(notice that it could need long time to run)
results <- estProfileWithGBPCR(snpName=sample$snpName, chr=sample$chr, position=sample$position, call=sample$call, rawLogratio=sample$rawLogratio, estLogratio=sample$estLogratio, thrHist=thrHist, chrToBeAnalyzed=7, maxProbeNumber=14000)
UPD <- results$estUPD
UPD[UPD == 0] <- NA
UPD[UPD == 1] <- 0
plot(sample$position[sample$chr == 7],sample$rawLogratio[sample$chr == 7])
points(sample$position[sample$chr == 7],sample$estLogratio[sample$chr == 7],col=3,type='l',lwd=3)
points(sample$position[sample$chr == 7],cna2logCn(results$estCNA[sample$chr == 7]),col=2,type='l',lwd=2)
points(sample$position[sample$chr == 7],UPD[sample$chr == 7],col=5,type='l',lwd=2)
legend(x="bottomleft",legend=c('mBPCR','CNAs','IBD/UPD'),lty=c(1,1,1),col=c(3,2,5))

#EXAMPLE 3
###In sample NA10851_UPD_20, the portion of chromosome 7 analized in EXAMPLE 1 has a region of copy-neutral LOH
###corresponding to the region of loss found in NA10851_LOH_20
###import the 250K nsp data of sample NA10851_UPD_20
path <- paste(getwd(), "/data/NA10851_UPD_20.dat",sep='')
sample <- importGenomicData(path, NRowSkip=1)
###set the parameters as in EXAMPLE 1
chr1 <- sample$chr
chr1[chr1==7][-(1:500)] <- 8
pHetData <- xPrior(typeArray='Affy250Knsp', race='CEU')
pHet1 <- array(dim=length(chr1))
pHet1[chr1==7] <- pHetData$pHet[pHetData$chrPHet == 7][1:500] 
load(paste(getwd(),'/data/paramHist20.RData',sep=''))
thrHist <- createThr(paramHist)
###estimation of the profile of the part of interest of chromosome 7
results <- estProfileWithGBPCR(snpName=sample$snpName, chr=chr1, position=sample$position, call=sample$call, rawLogratio=sample$rawLogratio, estLogratio=sample$estLogratio, thrHist=thrHist, chrToBeAnalyzed=7, maxProbeNumber=1000, pHet=pHet1, kMax=10)
##plot the estimated profile 
UPD <- results$estUPD
UPD[UPD == 0] <- NA
UPD[UPD == 1] <- 0
plot(sample$rawLogratio[chr1 == 7])
points(sample$estLogratio[chr1 == 7],col=3,type='l',lwd=3)
points(cna2logCn(results$estCNA[chr1 == 7]),col=2,type='l',lwd=2)
points(UPD[chr1 == 7],col=5,type='l',lwd=2)
legend(x="bottomleft",legend=c('mBPCR','CNAs','IBD/UPD'),lty=c(1,1,1),col=c(3,2,5))
```

---

[Package Index]
