# Supplementary material for: An integrated Bayesian analysis of LOH and copy number data
Source: BMC Bioinformatics. 2010 Jun 15;11:321. doi: 10.1186/1471-2105-11-321 (PMC2912301; doi:10.1186/1471-2105-11-321)
Supplement: Additional file 1 — gBPCR source code. This zipped file contains the source code of the gBPCR algorithm in R, including help files, sample data and examples. [file 1471-2105-11-321-S1.ZIP › gBPCRsource_code/html/gBPCR-internal.html]

R: Internal gBPCR functions

|  |  |
| --- | --- |
| gBPCR-internal {gBPCR} | R Documentation |

## Internal gBPCR functions

### Description

Internal functions of package gBPCR.

### Usage

```
computeGBPCR(y, pTilde, qTilde, epsilon, paramL, kMax=50, pUPD=0.0001, thr1="01", thr2="01")
computeLA0VectW(y, pTilde, qTilde, epsilon, paramL, pUPD = 0.0001)
computePCRegW(y, lL, lR, epsilon, paramL, pTilde, qTilde, pUPD=0.0001, kMax=50, thr1="01", thr2="01")
computeRecursionsGBPCR(lA0, n, kMax=50)
defCallRate(call, callNC, estLogratio, thrHist) 
indexLA0(r, c, n)
lJointDistrW(y, i, j, pTilde, qTilde, epsilon, paramL, pUPD=0.0001)
logAddVect(z)
paramAdj(paramL, epsilon, rate)
zPrior(estLogratio, rhoSquare, sigmaSquare, thrHist)
```

### Details

These functions are not to be called directly by the user

---

[Package Index]
