# Supplementary material for: An integrated Bayesian analysis of LOH and copy number data
Source: BMC Bioinformatics. 2010 Jun 15;11:321. doi: 10.1186/1471-2105-11-321 (PMC2912301; doi:10.1186/1471-2105-11-321)
Supplement: Additional file 1 — gBPCR source code. This zipped file contains the source code of the gBPCR algorithm in R, including help files, sample data and examples. [file 1471-2105-11-321-S1.ZIP › gBPCRsource_code/html/genAber2state.html]

R: Genomic aberration conversion from abbreviations to state values

|  |  |
| --- | --- |
| genAber2state {gBPCR} | R Documentation |

## Genomic aberration conversion from abbreviations to state values

### Description

Function that converts the genomic aberrations (in terms of their abbreviations) in their corresponding state values (used in the algorithm gBPCR).

### Usage

```
  genAber2state(genAber)
```

### Arguments

|  |  |
| --- | --- |
| `genAber` | array containing the genomic aberrations. The genomic aberrations are codified as following: `A` (high amplification), `G` (gain), `N` (normal state), `L` (loss of one copy), `HD` (homozygous deletion, i.e. loss of two copies), `IBD/UPD` (copy-neutral LOH). |

### Value

A numeric array with elements equal to: `3` at `A` (high amplification), `5` at `G` (gain), `1` at `N` (normal state),
`2` at `L` (loss of one copy), `4` at `HD` (homozygous deletion, i.e. loss of two copies), `0` at `IBD/UPD` (copy-neutral LOH).

### Note

The inverse function is called `state2genAber`.

### See Also

`state2genAber`,`stateConversion`

### Examples

```
##let us define an array of genomic aberrations (in terms of their abbreviations) 
genAber <- c(array("G", dim=200), array("IBD/UPD", dim=50), array("A", dim=100), array("N", dim=200))
##now we convert the genomic aberrations by using genAber2state and we plot them
plot(genAber2state(genAber))
```

---

[Package Index]
