# Supplementary material for: An integrated Bayesian analysis of LOH and copy number data
Source: BMC Bioinformatics. 2010 Jun 15;11:321. doi: 10.1186/1471-2105-11-321 (PMC2912301; doi:10.1186/1471-2105-11-321)
Supplement: Additional file 1 — gBPCR source code. This zipped file contains the source code of the gBPCR algorithm in R, including help files, sample data and examples. [file 1471-2105-11-321-S1.ZIP › gBPCRsource_code/html/importGenomicData.html]

R: Import genomic data

|  |  |
| --- | --- |
| importGenomicData {gBPCR} | R Documentation |

## Import genomic data

### Description

Function to import the genomic data from a tab delimited file.

### Usage

```
  importGenomicData(path, NRowSkip, ifLogRatio=1)
```

### Arguments

|  |  |
| --- | --- |
| `path` | path of the tab delimited file containing the genomic data of the sample. The file must contain a table of six columns, where in the first column there are the names of the probes (snpName), in the second one, the chromosome to which each probe belongs (the possible values of the chromosomes are: the integers from 1 to 22, 'X' and 'Y'), in the third one, the phisical positions of the probes, in the fourth one, the copy number data, in the fifth one the estimated copy numbers and in the sixth one the genotyping data. |
| `NRowSkip` | number of rows to skip in the file, before the table. The names of the columns are to be skipped. |
| `ifLogRatio` | denotes whether the raw and estimated copy numbers are in log2ratio scale. By default, they are considered in log2ratio scale, otherwise (`ifLogRatio=0`) they are transformed in log2ratio scale. |

### Value

A list containing:

|  |  |
| --- | --- |
| `snpName` | an array containing the names of the probes |
| `chr` | an array containing the name of the chromosome to which each probe belongs |
| `position` | an array containing the physical position of each probe |
| `rawLogratio` | array containing the log2ratio of the raw copy number data |
| `estLogratio` | array containing the estimated log2ratio of the copy number as a piecewise constant function (preferably by using mBPCR) |
| `call` | array containing the genotyping data |

### Examples

```
###Before using the following commands, set "gBPCR" as working directory

###import the 250K nsp data of sample NA10851_LOH_20
path <- paste(getwd(), "/data/NA10851_LOH_20.dat",sep='')
sample <- importGenomicData(path, NRowSkip=1)
###we plot the raw and estimated log2ratio of the copy number of chromosome 7
plot(sample$position[sample$chr == 7], sample$rawLogratio[sample$chr == 7], pch='.', cex=2, col='grey')
points(sample$position[sample$chr == 7], sample$estLogratio[sample$chr == 7], type="l", col=2)
```

---

[Package Index]
