# Supplementary material for: An integrated Bayesian analysis of LOH and copy number data
Source: BMC Bioinformatics. 2010 Jun 15;11:321. doi: 10.1186/1471-2105-11-321 (PMC2912301; doi:10.1186/1471-2105-11-321)
Supplement: Additional file 1 — gBPCR source code. This zipped file contains the source code of the gBPCR algorithm in R, including help files, sample data and examples. [file 1471-2105-11-321-S1.ZIP › gBPCRsource_code/html/logAdd.html]

R: Overflow-safe computation of the logarithm of a sum

|  |  |
| --- | --- |
| logAdd {mBPCR} | R Documentation |

## Overflow-safe computation of the logarithm of a sum

### Description

Function to compute the logarithm of a sum of small numbers, avoiding overflow.

### Usage

```
  logAdd(x)
```

### Arguments

|  |  |
| --- | --- |
| `x` | array or matrix containing the logarithm of the terms of the sum. If `x` is a matrix, the function return the results by column. |

### Value

If `x` is an array, the function returns *log(sum\_i(e^x[i]))*, otherwise it returns an array containing the results by column.

### Examples

```
x <- log(c(0.0001, 0.0003, 0.000006))
y <- logAdd(x) 
##verification that the computation is correct
z <- sum(c(0.0001, 0.0003, 0.000006))
z
exp(y)
```

---

[Package Index]
