# Supplementary material for: An integrated Bayesian analysis of LOH and copy number data
Source: BMC Bioinformatics. 2010 Jun 15;11:321. doi: 10.1186/1471-2105-11-321 (PMC2912301; doi:10.1186/1471-2105-11-321)
Supplement: Additional file 1 — gBPCR source code. This zipped file contains the source code of the gBPCR algorithm in R, including help files, sample data and examples. [file 1471-2105-11-321-S1.ZIP › gBPCRsource_code/html/logCn2cna.html]

R: Copy number aberration (CNA) conversion from log2ratio symbols to CNA abbreviations

|  |  |
| --- | --- |
| logCn2cna {gBPCR} | R Documentation |

## Copy number aberration (CNA) conversion from log2ratio symbols to CNA abbreviations

### Description

Function that converts the "symbolic" log2ratio values corresponding to the copy number aberrations (CNAs) in CNA abbreviations.

### Usage

```
  logCn2cna(logCn)
```

### Arguments

|  |  |
| --- | --- |
| `logCn` | array containing the "symbolic" log2ratio values of the copy number aberrations (CNAs). The CNAs are codified as following: `2.5` (high amplification), `1` (gain), `0` (normal copy number), `-1` (loss of one copy), `-2.5` (homozygous deletion, i.e. loss of two copies). |

### Value

An array with elements equal to: `A` at `2.5` (high amplification), `G` at `1` (gain), `N` at `0` (normal copy number),
`L` at `-1` (loss of one copy), `HD` at `-2.5` (homozygous deletion, i.e. loss of two copies).

### Note

The inverse function is called `cna2logCn`.

### See Also

`cna2logCn`,`stateConversion`

### Examples

```
##let us define an array of "symbolic" log2ratio values corresponding to the copy number aberrations  
logCn <- c(array(2.5, dim=20), array(0, dim=5), array(-1, dim=10), array(-2.5, dim=20))
##now we convert the "symbolic" log2ratio values by using logCn2cna
logCn2cna(logCn)
```

---

[Package Index]
