# Supplementary material for: An integrated Bayesian analysis of LOH and copy number data
Source: BMC Bioinformatics. 2010 Jun 15;11:321. doi: 10.1186/1471-2105-11-321 (PMC2912301; doi:10.1186/1471-2105-11-321)
Supplement: Additional file 1 — gBPCR source code. This zipped file contains the source code of the gBPCR algorithm in R, including help files, sample data and examples. [file 1471-2105-11-321-S1.ZIP › gBPCRsource_code/html/plotEstProfileGBPCR.html]

R: Plot the estimated profile of genomic aberrations

|  |  |
| --- | --- |
| plotEstProfileGBPCR {gBPCR} | R Documentation |

## Plot the estimated profile of genomic aberrations

### Description

Function to plot the estimated profiles of genomic aberrations.

### Usage

```
  plotEstProfileGBPCR(sampleName='', chr, position, rawLogratio, chrToBePlotted, estCNA, estUPD, maxProbeNumber,
                 legendPosition='bottomleft', hg=17)
```

### Arguments

|  |  |
| --- | --- |
| `sampleName` | name of the sample, if the user wants to put it in the title of the graph |
| `chr` | array containing the name of the chromosome to which each probe belongs. The possible values of the elements of `chr` are: the integers from 1 to 22, 'X' and 'Y'. |
| `position` | array containing the physical position of each probe |
| `rawLogratio` | array containing the log2ratio of the raw copy number data |
| `chrToBePlotted` | array containing the name of the estimated chromosomes, that the user wants to plot. The possible values of the chromosomes are: the integers from 1 to 22, 'X' and 'Y'. |
| `estCNA` | array containing the estimated copy number aberrations (CNAs) by using gBPCR (it has the same length of `position` and `chr`). The CNAs are codified as following: `A` (high amplification), `G` (gain), `N` (normal copy number), `L` (loss of one copy), `HD` (homozygous deletion, i.e. loss of two copies). |
| `estUPD` | array containing the estimated regions of IBD/UPD by using gBPCR (it has the same length of `position` and `chr`). The elements of the array are: `1`, if there is a copy-neutral LOH in the position, and `0`, otherwise. |
| `maxProbeNumber` | maximum number of probes that a chromosome (or arm of a chromosome) can have to be analyzed. The procedure of profile estimation needs the computation of an array of length *(length(chromosome)+1)\*(length(chromosome)+2)/2*. To be sure to have set this parameter correctly, try to create the array `A <- array(1, dim=(maxProbeNumber+1)*(maxProbeNumber+2)/2)`, before starting with the estimation procedure. |
| `legendPosition` | string containing the position of the legend in the plot. The possible values are the same used in the function `plot`. |
| `hg` | reference of human genome used for the annotation of the SNPs. The following value are supported: 17 and 18. |

### Details

The function plots the estimated profiles of genomic aberrations of the chromosomes of `chrToBePlotted`, separately.

### Examples

```
###Before using the following commands, set "gBPCR" as working directory

###import the 250K nsp data of sample NA10851_LOH_20
path <- paste(getwd(), "/data/NA10851_LOH_20.dat",sep='')
sample <- importGenomicData(path, NRowSkip=1)
###we select only the data belonging to the first part of chromosome 7
chr1 <- sample$chr
chr1[chr1==7][-(1:500)] <- 8
pHetData <- xPrior(typeArray='Affy250Knsp', race='CEU')
pHet1 <- array(dim=length(chr1))
pHet1[chr1==7] <- pHetData$pHet[pHetData$chrPHet == 7][1:500] 
load(paste(getwd(),'/data/paramHist20.RData',sep=''))
thrHist <- createThr(paramHist)
###estimation of the profile of the part of interest of chromosome 7
results <- estProfileWithGBPCR(snpName=sample$snpName, chr=chr1, position=sample$position, call=sample$call, rawLogratio=sample$rawLogratio, estLogratio=sample$estLogratio, thrHist=thrHist, chrToBeAnalyzed=7, maxProbeNumber=1000, pHet=pHet1, kMax=10)
###plot the corresponding estimated profiles
plotEstProfileGBPCR(sampleName='NA10851_LOH_20', chr1, position=sample$position, rawLogratio=sample$rawLogratio, chrToBePlotted=7, estCNA=results$estCNA, estUPD=results$estUPD, maxProbeNumber=1000, legendPosition='bottomleft')
```

---

[Package Index]
