# Supplementary material for: An integrated Bayesian analysis of LOH and copy number data
Source: BMC Bioinformatics. 2010 Jun 15;11:321. doi: 10.1186/1471-2105-11-321 (PMC2912301; doi:10.1186/1471-2105-11-321)
Supplement: Additional file 1 — gBPCR source code. This zipped file contains the source code of the gBPCR algorithm in R, including help files, sample data and examples. [file 1471-2105-11-321-S1.ZIP › gBPCRsource_code/html/state2genAber.html]

R: Genomic aberration conversion from state values to abbreviations

|  |  |
| --- | --- |
| state2genAber {gBPCR} | R Documentation |

## Genomic aberration conversion from state values to abbreviations

### Description

Function that converts the state values (used in the algorithm gBPCR) corresponding to the genomic aberrations in their abbreviations.

### Usage

```
  state2genAber(state)
```

### Arguments

|  |  |
| --- | --- |
| `state` | array containing the state values (used in the algorithm gBPCR) of the genomic aberrations. The genomic aberrations are codified as following: `3` (high amplification), `5` (gain), `1` (normal state), `2` (loss of one copy), `4` (homozygous deletion, i.e. loss of two copies), `0` (copy-neutral LOH). |

### Value

An array with elements equal to: `A` at `3` (high amplification), `G` at `5` (gain), `N` at `1` (normal state),
`L` at `2` (loss of one copy), `HD` at `4` (homozygous deletion, i.e. loss of two copies), `IBD/UPD` at `0` (copy-neutral LOH).

### Note

The inverse function is called `genAber2state`.

### See Also

`genAber2state`,`stateConversion`

### Examples

```
##let us define an array of state values corresponding to the genomic aberrations  
state <- c(array(5, dim=20), array(0, dim=5), array(3, dim=10), array(1, dim=20))
##now we convert the state values by using state2genAber
state2genAber(state)
```

---

[Package Index]
