# Supplementary material for: An integrated Bayesian analysis of LOH and copy number data
Source: BMC Bioinformatics. 2010 Jun 15;11:321. doi: 10.1186/1471-2105-11-321 (PMC2912301; doi:10.1186/1471-2105-11-321)
Supplement: Additional file 1 — gBPCR source code. This zipped file contains the source code of the gBPCR algorithm in R, including help files, sample data and examples. [file 1471-2105-11-321-S1.ZIP › gBPCRsource_code/html/stateConversion.html]

R: Genomic aberration conversion from state values to CNAs and copy-neutral LOH regions

|  |  |
| --- | --- |
| stateConversion {gBPCR} | R Documentation |

## Genomic aberration conversion from state values to CNAs and copy-neutral LOH regions

### Description

Function that converts the genomic aberrations (in terms of the state values used in the algorithm) in two arrays: one corresponding
to the copy number aberrations (CNAs) and the other corresponding to the presence of a copy-neutral LOH.

### Usage

```
  stateConversion(state)
```

### Arguments

|  |  |
| --- | --- |
| `state` | array containing the state values (used in the algorithm gBPCR) of the genomic aberrations. The genomic aberrations are codified as following: `3` (high amplification), `5` (gain), `1` (normal state), `2` (loss of one copy), `4` (homozygous deletion, i.e. loss of two copies), `0` (copy-neutral LOH). |

### Value

A list containing:

|  |  |
| --- | --- |
| `logCn` | array containing the "symbolic" log2ratio values of the copy number aberrations (CNAs). The CNAs are codified as following: `2.5` (high amplification), `1` (gain), `0` (normal copy number), `-1` (loss of one copy), `-2.5` (homozygous deletion, i.e. loss of two copies). |
| `upd` | array containing the copy-neutral LOH regions. The elements of the array are: `1`, if there is a copy-neutral LOH in the position, and `0`, otherwise. |

### See Also

`state2genAber`,`logCn2cna`

### Examples

```
##let us define an array of state values corresponding to the genomic aberrations  
state <- c(array(5, dim=200), array(0, dim=50), array(3, dim=100), array(1, dim=200))
##now we convert the state values by using stateConversion and we plot them
results <- stateConversion(state)
plot(results$logCn,col=2)
results$upd[results$upd == 0] <- NA
results$upd[results$upd == 1] <- 0
points(results$upd,col=5)
legend(x='bottomleft',legend=c('CNAs','IBD/UPD'),lty=c(1,1),col=c(2,5))
```

---

[Package Index]
