# Supplementary material for: An integrated Bayesian analysis of LOH and copy number data
Source: BMC Bioinformatics. 2010 Jun 15;11:321. doi: 10.1186/1471-2105-11-321 (PMC2912301; doi:10.1186/1471-2105-11-321)
Supplement: Additional file 1 — gBPCR source code. This zipped file contains the source code of the gBPCR algorithm in R, including help files, sample data and examples. [file 1471-2105-11-321-S1.ZIP › gBPCRsource_code/html/xPrior.html]

R: Import the probabilities of heterozygosity

|  |  |
| --- | --- |
| xPrior {gBPCR} | R Documentation |

## Import the probabilities of heterozygosity

### Description

Function that import the probabilities of heterozygosity of the SNPs from the annotation files in the package.

### Usage

```
  xPrior(typeArray='Affy250Knsp', race='CEU')
```

### Arguments

|  |  |
| --- | --- |
| `typeArray` | denotes the type of microarray used (currently only the value `Affy250Knsp` is supported) |
| `race` | denotes the race of the patient. Currently the following values are supported: `CEU` (Utah residents with Northern and Western European ancestry from the CEPH collection), `CHB` (Han Chinese in Beijing, China), `JPT` (Japanese in Tokyo, Japan), `YRI` (Yoruban in Ibadan, Nigeria). |

### Value

A list with two fields:

|  |  |
| --- | --- |
| `pHet` | array containing the probability of heterozygosity of each SNP |
| `chrPHet` | array containing the name of the chromosome to which each of the probes belongs. The possible values of the elements of `chr` are: the integers from 1 to 22, 'X' and 'Y'. |

### Note

Currently, the annotation files in the package regard only the Affymetrix GeneChip Human Mapping 250K NspI.

### See Also

`estProfileWithGBPCR`

### Examples

```
###Before using the following commands, set "gBPCR" as working directory 

##import the probabilities of heterozygosity of the SNPs in the Affymetrix GeneChip Human Mapping 250K NspI for the 'CHB' race 
pHetData <- xPrior(typeArray='Affy250Knsp', race='CHB')
##plot the probabilities of heterozygosity of the SNPs in chromosome 7
plot(pHetData$pHet[pHetData$chrPHet == 7])
```

---

[Package Index]
