# Supplementary material for: An integrated Bayesian analysis of LOH and copy number data
Source: BMC Bioinformatics. 2010 Jun 15;11:321. doi: 10.1186/1471-2105-11-321 (PMC2912301; doi:10.1186/1471-2105-11-321)
Supplement: Additional file 1 — gBPCR source code. This zipped file contains the source code of the gBPCR algorithm in R, including help files, sample data and examples. [file 1471-2105-11-321-S1.ZIP › gBPCRsource_code/html/createThr.html]

R: Estimate the thresholds of the copy number aberrations

|  |  |
| --- | --- |
| createThr {gBPCR} | R Documentation |

## Estimate the thresholds of the copy number aberrations

### Description

Function to estimate the thresholds of the copy number aberrations which are used to compute the prior of the copy number aberrations.

### Usage

```
  createThr(paramHist)
```

### Arguments

|  |  |
| --- | --- |
| `paramHist` | list containing two fields: `mu` and `s2`. `mu` is a numeric array containing the mean values of the normal distributions corresponding to the loss, normal and gain states (in the multivariate normal distribution of the estimated log2ratio values). `s2` is a numeric array containing the variances of the normal distributions corresponding to the loss, normal and gain states (in the multivariate normal distribution of the estimated log2ratio values). |

### Details

The function computes the thresholds of the copy number aberrations in the following way:
  
thr[1] <- mu[1]-3\*sqrt(s2[1])
  
thr[2] <- mu[2]-3\*sqrt(s2[2])
  
thr[3] <- mu[2]+3\*sqrt(s2[2])
  
thr[4] <- mu[3]+3\*sqrt(s2[3])
  
The thresholds have the following meaning:
  
- log2ratio ≤ thr[1] : homozygous deletion
  
- thr[1] < log2ratio ≤ thr[2] : loss
  
- thr[2] < log2ratio ≤ thr[3] : normal
  
- thr[3] < log2ratio ≤ thr[4] : gain
  
- log2ratio > thr[4] : high amplification

### Value

An array containg the thresholds explained above.

### References

Rancoita, P. M. V., Hutter, M., Bertoni, F., Kwee, I. (2010). An integrated Bayesian analysis of LOH and copy number data. Submitted.
  
http://www.idsia.ch/~paola/gBPCR

### See Also

`estProfileWithGBPCR`

### Examples

```
###Before using the following commands, set "gBPCR" as working directory

###import the parameters of the histogram of the log2ratios corresponding to sample NA10851_LOH_20
load(paste(getwd(), "/data/paramHist20.RData",sep=''))
thrHist <- createThr(paramHist)
```

---

[Package Index]
