# Supplementary material for: An integrated Bayesian analysis of LOH and copy number data
Source: BMC Bioinformatics. 2010 Jun 15;11:321. doi: 10.1186/1471-2105-11-321 (PMC2912301; doi:10.1186/1471-2105-11-321)
Supplement: Additional file 2 — Supplementary material. This file contains: 1) the description of the estimation of the parameters of the likelihood, 2) the explanation of the estimation of density of the estimated log2ratio levels, 3) explicit formulae of some quantities employed in the dynamic programming used to implement our method, 4) the explanation of an algorithm for the determination of the maxima of a multimodal function, 5) detailed description of the results obtained on simulated data, 6) some supplementary tables and 7) some supplementary figures. [file 1471-2105-11-321-S2.PDF]

Supplementary material

S.1 Estimation of the parameters of the likelihood

To estimate the parameters of the likelihood we needed paired normal-cancer samples, since they are related to the probability of detecting a certain homozygous status in a cancer cell, given the corresponding one in a normal cell of the same sample, under some copy number event. We used 13 breast cancer cell line samples of [27,28], suitable for our purpose: HCC1143, HCC1143 BL, HCC1143M6, HCC1143M7, HCC1143M8, HCC1143M9, HCC38, HCC38 BL march, HCC38 BL may, HCC38M6, HCC38M7, HCC38M8, HCC38M9 (the "BL" in cell line names refers to B-lymphoblast and, in each cell line pair, the BL cell line is used as the normal control). The data were obtained with the XbaI mapping array 130 (Affymetrix, Inc., Santa Clara, CA) and the preprocessing of the data were performed with dChip [16]. To obtain a better normalization of the data, we analyzed them together with other 5 normal cell lines in the dataset: HCC1187 BL, HCC1395 BL, HCC1599 BL, HCC1937 BL, HCC2218 BL.

The following two probabilities are related to a normal copy number event and represent errors due to the detection of $NoCall$ instead of $NHet$ and Het, respectively,

$$\begin{aligned} \mathrm{P}(Y_i = NoCall \,|\, X_i^N = Hom,\, \widetilde{Z}_i = 0) &= \delta_1 \\ \mathrm{P}(Y_i = NoCall \,|\, X_i^N = Het,\, \widetilde{Z}_i = 0) &= \delta_2. \end{aligned}$$

To estimate them, we used chromosome 1 of two replicates of the normal cell line HCC38 BL. In this chromosome we did not find any SNP with homozygous call in one sample and heterozygous call in the other one, thus we assumed that the detected genotypes (different from $NoCall$) were all correct. Instead, we found 27 $NoCall$ SNPs in both samples, so that we eliminated them from the analysis. The estimated parameters were:

$$\begin{aligned} \hat{\delta}_1 &= \frac{\#\{\text{ SNPs homozygous in one sample and } NoCall \text{ in the other}\}}{\#\{\text{homozygous SNPs in at least one sample}\}} \\ \hat{\delta}_2 &= \frac{\#\{\text{ SNPs heterozygous in one sample and } NoCall \text{ in the other}\}}{\#\{\text{heterozygous SNPs in at least one sample}\}} \end{aligned}$$

Regarding the probabilities $\mathrm{P}(Y_i = Het \,|\, X_i^N = Hom,\, \widetilde{Z}_i = 0)$ and $\mathrm{P}(Y_i = NHet \,|\, X_i^N = Het,\, \widetilde{Z}_i = 0)$, we set them as the genotyping detection error.

Other probabilities are related to errors due also to the presence of a subpopulation of cells in the tumor sample (normal cells or tumor cells in another stage of the disease). Hence, to estimate them, we used the human breast carcinoma cell lines HCC1143 and HCC38, because we had samples containing 0, 60, 70, 80, 90, 100% of tumor cells for each cell line, from [27,28]. We defined some regions of amplification, loss and homozygous deletion on the basis of the regions of copy number changes indicated by [27,28]. For each region, we looked at the copy number value of the SNPs to better identify the start/end SNP of the aberrant region, since, in [27,28], they were only denoted by the corresponding cytobands. Finally, the estimations of the probabilities were performed using the maximum likelihood estimators averaging over all

the samples of the same cell line with some percentage of tumor cells, for z = -2, -1, 2,

$$\hat{\mathrm{P}}(Y_i = Het \,|\, X_i^N = x,\, \widetilde{Z}_i = z) \;=\; \frac{1}{5\sum_c n_c} \sum_{\substack{c\in\{\mathrm{HCC38},\\ \mathrm{HCC1143}\}}} \sum_{h=1}^{5} \sum_{j=1}^{n_c} (SNP_{j,c,h}^2 + SNP_{j,c,h})$$

$$\hat{\mathrm{P}}(Y_i = NoCall \,|\, X_i^N = x,\, \widetilde{Z}_i = z) \;=\; \frac{1}{5\sum_c n_c} \sum_{\substack{c\in\{\mathrm{HCC38},\\ \mathrm{HCC1143}\}}} \sum_{h=1}^{5} \sum_{j=1}^{n_c} (SNP_{j,c,h}^2 - SNP_{j,c,h})$$

where n_c is the total number of the SNPs in the regions with $\{X_i^N = x,\, \widetilde{Z}_i = z\}$ of cell line c (c= HCC38, HCC1143), and $SNP_{j,c,h}$ is a value assign to j^{th} SNP of sample h of cell line c, based on its homozygous status value $Y_{j,c,h}$:

$$SNP_{j,c,h} = \begin{cases} -1 & \text{if } Y_{j,c,h} = NoCall \\ 0 & \text{if } Y_{j,c,h} = NHet \\ 1 & \text{if } Y_{j,c,h} = Het. \end{cases}$$

It remains to estimate the following probabilities related to the IBD/UPD events,

$$\mathrm{P}(Y_i = NHet \,|\, \widetilde{Z}_i = 0,\, \widetilde{U}_i = 1) \;=\; \delta_3$$
$$\mathrm{P}(Y_i = NoCall \,|\, \widetilde{Z}_i = 0,\, \widetilde{U}_i = 1) \;=\; \delta_4,$$

that we added in Model 2. For their estimation, we used 11 IBD/UPD regions previously found by us on 5 samples of patients with hairy cell leukemia [34] and on the B-cell lymphoma cell line KARPAS-422 (unpublished). The data were obtained with the GeneChip Human Mapping 250K NspI (Affymetrix, Santa Clara, CA, USA). The genotype calls were calculated with the BRLMM [26] algorithm using 46 Caucasian normal female samples of the HapMap Project as reference samples and the raw copy number data were retrieved using CNAT 4.01 [36]. All IBD/UPD regions were detected by dChip [16], from the genotype calls estimated by BRLMM. Their width was between 3Mb and 100Mb (covering from 300 to 9800 SNPs), so that they were large enough to be really considered IBD/UPD regions.

We computed the estimators for δ_3 and δ_4 simply using the frequency of $NHet$ and $NoCall$ in the selected IBD/UPD regions, respectively. We found $\hat{\delta}_4$ equal to the arithmetic mean of $\hat{\delta}_1$ and $\hat{\delta}_2$ (the errors of detecting a $NoCall$ in a normal region instead of $NHet$ or Het, respectively), which is a realistic result since we can have both homozygous and heterozygous SNPs in an IBD/UPD region.

S.2 Estimation of the estimated $\log_2$ratio levels density

To estimate the density of the estimated $\log_2$ratio levels as a mixture of three or four normal distributions, at first we tried some existing methods. Unfortunately, the functions, available in R for this purpose, frequently do not converge and are very sensitive to the initial values. Hence, we decided to first estimate roughly each peak of the density separately, and then use them as guess values in an algorithm for the estimation of a normal mixture density. The steps for the rough estimation of the parameters of the peak distributions are the following:

1. make an histogram of the $\log_2$ratio values using a high number of bins (for example, 80 or 100);

2. estimate the position of the highest peak (which corresponds to $CN = 2$) and select all the intervals around this peak whose density is estimated greater than $e^{-2} \times$ "value of the highest peak" (in a normal distribution, this means that the points have a distance from the mean greater than 2 times the standard deviation);

3. use the points inside the selected intervals to estimate the mean (μ_2) and the variance (σ_2^2) of a normal distribution, in the classical way;

4. select the intervals of the histogram lower than $\hat{\mu}_2 - 3\hat{\sigma}_2$ and repeat step 2 and 3 to find the mean μ_1 and the variance σ_1^2 of the peak corresponding to $CN = 1$;

5. select the intervals of the histogram greater than $\hat{\mu}_2 + 3\hat{\sigma}_2$ and repeat step 2 and 3 to find the mean μ_3 and the variance σ_3^2 of the highest peak of the interval which corresponds to $CN = 3$;

6. select the intervals of the histogram greater than $\hat{\mu}_3 + 3\hat{\sigma}_3$ and repeat step 2 and 3 to find the mean μ_4 and the variance σ_4^2 of the peak corresponding to $CN = 4$.

If there is only one peak corresponding to $CN = \{3, 4\}$, we do not need step 6. and the mean and variance estimated at step 5. are called μ_4 and σ_4^2, respectively.

S.3 Dynamic programming

Since we assume a uniform prior distribution for the boundaries (see Section "Methods"), we can use the same recursion employed in mBPCR for the computation of the posterior probabilities (see [14]). Then, for computational purpose, we need only to make explicit the formula of A^0_{ij} (the probability of $\boldsymbol{Y}_{ij} = \boldsymbol{y}_{ij}$ given cn and knowing that $\boldsymbol{Y}_{ij}$ belongs to only one segment called p).

First, let us consider Model 1. Conditioning with respect to Z_p and using the independence of the data points Y_s ($s = i+1, \ldots, j$) given Z_p, we obtain

$$\begin{aligned}
\mathrm{A}^0_{ij} &= p(\boldsymbol{y}_{ij} \,|\, K_{ij} = 1,\, T_{p-1} = i,\, T_p = j,\, cn) \\
&= \sum_{z \in \{-2,-1,0,2\}} p(\boldsymbol{y}_{ij},\, Z_p = z \,|\, K_{ij} = 1,\, T_{p-1} = i,\, T_p = j,\, cn) \\
&= \sum_{z \in \{-2,-1,0,2\}} p(\boldsymbol{y}_{ij} \,|\, Z_p = z,\, K_{ij} = 1,\, T_{p-1} = i,\, T_p = j) \mathrm{G}_{i,j}(z) \\
&= \sum_{z \in \{-2,-1,0,2\}} \prod_{s=i+1}^{j} p(y_s \,|\, \widetilde{Z}_s = z) \mathrm{G}_{i,j}(z),
\end{aligned} \tag{S.1}$$

where $\mathrm{G}_{i,j}(z) = \mathrm{P}(Z_p = z \,|\, K_{ij} = 1,\, T_{p-1} = i,\, T_p = j,\, cn)$. The computation of $\mathrm{G}_{i,j}(z)$, given cn, can be done by using Bayes Theorem and the equivalence between the events $\{K_{ij} = 1\}$

and $\{\widetilde{Z}_s = Z_p = z$ for all $s = i+1, \ldots, j$, for some $z \in \{-2, -1, 0, 2\}\}$,

$$
\begin{aligned}
\mathrm{G}_{i,j}(z) &= \mathrm{P}(Z_p = z \,|\, K_{ij} = 1, T_{p-1} = i, T_p = j, cn) \\
&= \mathrm{P}\left(\bigcap_{s=i+1}^{j} \{\widetilde{Z}_s = z\} \,|\, K_{ij} = 1, T_{p-1} = i, T_p = j, cn\right) \\
&= \frac{\mathrm{P}(\bigcap_{s=i+1}^{j}\{\widetilde{Z}_s = z\}, K_{ij} = 1 \,|\, T_{p-1} = i, T_p = j, cn)}{\mathrm{P}(K_{ij} = 1 \,|\, T_{p-1} = i, T_p = j, cn)} \\
&= \frac{\mathrm{P}(\bigcap_{s=i+1}^{j}\{\widetilde{Z}_s = z\} \,|\, cn)}{\sum_{z\in\{-2,-1,0,2\}} \mathrm{P}(\bigcap_{s=i+1}^{j}\{\widetilde{Z}_s = z\} \,|\, cn)},
\end{aligned}
\tag{S.2}
$$

where,

$$
\mathrm{P}\left(\bigcap_{s=i+1}^{j} \{\widetilde{Z}_s = z\} \,\middle|\, cn\right) = \prod_{\hat{t}_{\tilde{q}}^{cn} \in \mathcal{T}_{i+1,j}} \mathrm{P}(Z_{\hat{t}_{\tilde{q}}^{cn}} = z \,|\, cn),
\tag{S.3}
$$

with $\mathcal{T}_{i+1,j} = \{\hat{t}_{\tilde{q}}^{cn} \mid \hat{t}_{\tilde{q}}^{cn} \in [i+1, j),\ \tilde{q} = 1, \ldots, \hat{k}^{cn}\} \cup \min\{\hat{t}_{\tilde{q}}^{cn} \mid \hat{t}_{\tilde{q}}^{cn} \geq j,\ \tilde{q} = 1, \ldots, \hat{k}^{cn}\}$. In the previous formulas, $\hat{k}^{cn}$ and $\hat{\boldsymbol{t}}^{cn}$ represent the estimated number of segments and boundaries of the $\log_2$ratio profile, respectively. Notice that the prior probability of $\widetilde{\boldsymbol{Z}}_{ij}$ is based on the copy number estimation and that only the $\widetilde{Z}_s$ belonging to different segments are independent. Therefore, in Equation (S.3) we partitioned the interval $[i+1, j]$ in subintervals $\{I_q\}_q$, using the boundaries $\{\hat{t}_{\tilde{q}}^{cn} \mid \hat{t}_{\tilde{q}}^{cn} \in [i+1, j],\ \tilde{q} = 1, \ldots, \hat{k}^{cn}\}$. By definition, any interval I_q of $[i+1, j]$ is either equal or contained in a segment of the partition generated by the estimated $\log_2$ratio profile (called $I_{\tilde{q}}$, i.e. $(\hat{t}_{\tilde{q}-1}^{cn}, \hat{t}_{\tilde{q}}^{cn}]$). Therefore, $\mathrm{P}\left(\bigcap_{s\in I_q}\{\widetilde{Z}_s = z\} \,\middle|\, cn\right) = \mathrm{P}\left(Z_{\hat{t}_{\tilde{q}}^{cn}} = z \,|\, cn\right)$.

Using the dynamic programming, we can compute both $p(k \,|\, \boldsymbol{Y}, cn)$ and $p(\boldsymbol{t} \,|\, \boldsymbol{Y}, cn)$ and thus estimate k_0 and $\boldsymbol{t}^0$ with Equations (2) and (3). Finally, from Equations (5) and (S.1), it follows that the posterior distribution of Z_p can be written as

$$
\mathrm{P}(Z_p = z \,|\, \boldsymbol{y}, \hat{\boldsymbol{t}}, \hat{k}, cn) = \frac{\prod_{i=\hat{t}_{p-1}+1}^{\hat{t}_p} \mathrm{P}(y_i \,|\, \widetilde{Z}_i = z)\mathrm{G}_{\hat{t}_{p-1},\hat{t}_p}(z)}{\mathrm{A}^0_{\hat{t}_{p-1},\hat{t}_p}}, \qquad z = -2, -1, 0, 2,
\tag{S.4}
$$

and we can derive the MAP estimate of Z_p, for each $p = 1, \ldots, \hat{k}$.

If we consider Model 2, Equation (S.1) for the computation of the quantity A^0_{ij} becomes,

$$
\begin{aligned}
\mathrm{A}^0_{ij} &= \mathrm{P}(\underline{y}_{ij} \,|\, K_{ij} = 1, T_{p-1} = i, T_p = j, cn) \\
&= \sum_{z\in\{-2,-1,2\}} \prod_{s=i+1}^{j} \mathrm{P}(y_s \,|\, \widetilde{Z}_s = z)\mathrm{G}_{i,j}(z) + \left[\prod_{s=i+1}^{j} \mathrm{P}(y_s \,|\, \widetilde{Z}_s = 0, \widetilde{U}_s = 1)p_{upd} + \right. \\
&\quad \left. \prod_{s=i+1}^{j} \mathrm{P}(y_s \,|\, \widetilde{Z}_s = 0, \widetilde{U}_s = 0)(1 - p_{upd})\right] \mathrm{G}_{i,j}(0).
\end{aligned}
\tag{S.5}
$$

Moreover, for any $p = 1, \ldots, \hat{k}$, the posterior probability of W_p is

$$
\mathrm{P}(W_p = w \,|\, \underline{y}, \underline{\hat{t}}, \hat{k}, cn) = \mathrm{P}(Z_p = w \,|\, \underline{y}, \underline{\hat{t}}, \hat{k}, cn),
$$

for w = -2, -1, 2, and

$$\mathrm{P}(W_p = w \mid \underline{y}, \underline{\hat{t}}, \hat{k}, cn) = p_{upd}^{-w/3}(1-p_{upd})^{(3+w)/3}\frac{\prod_{i=\hat{t}_{p-1}+1}^{\hat{t}_p}\mathrm{P}(y_i \mid \widetilde{W}_i = w)\mathrm{G}_{\hat{t}_{p-1},\hat{t}_p}(0)}{\mathrm{A}^0_{\hat{t}_{p-1},\hat{t}_p}}, \tag{S.6}$$

for $w = -3$, 0, by using a derivation similar to the one of Equation (S.4).

Since we assume that there is no difference in the genotype detection between normal and gained regions, in Model 3 the probabilities $p(\boldsymbol{y} \mid Z_p = 0, \hat{\boldsymbol{t}}, \hat{k}, cn)$ and $p(\boldsymbol{y} \mid Z_p = 1, \hat{\boldsymbol{t}}, \hat{k}, cn)$ are equal. Therefore, the computation of A^0_{ij} in Equation (S.5) becomes

$$\begin{aligned}
\mathrm{A}^0_{ij} &= \mathrm{P}(\boldsymbol{y}_{ij} \mid K_{ij} = 1, T_{p-1} = i, T_p = j, cn) \\
&= \sum_{z\in\{-2,-1,2\}}\prod_{s=i+1}^{j}\mathrm{P}(y_s \mid \widetilde{Z}_s = z)\mathrm{G}_{i,j}(z) + \left[\prod_{s=i+1}^{j}\mathrm{P}(y_s \mid \widetilde{Z}_s = 0, \widetilde{U}_s = 1)p_{upd} + \right. \\
&\left.\prod_{s=i+1}^{j}\mathrm{P}(y_s \mid \widetilde{Z}_s = 0, \widetilde{U}_s = 0)(1-p_{upd})\right][\mathrm{G}_{i,j}(0) + \mathrm{G}_{i,j}(1)],
\end{aligned}$$

where now $G_{ij}(z)$ is calculated taking into account five classes of copy number events, instead of four as in Equation (S.2),

$$\mathrm{G}_{i,j}(z) = \frac{\mathrm{P}(\bigcap_{s=i+1}^{j}\{\widetilde{Z}_s = z\} \mid cn)}{\sum_{z=-2}^{2}\mathrm{P}(\bigcap_{s=i+1}^{j}\{\widetilde{Z}_s = z\} \mid cn)}.$$

Moreover, the posterior probabilities of $\{W_p = w\}$ are the same as before for $w = -2$, -1, 0, 2, while for $w = 1$,

$$\begin{aligned}
\mathrm{P}(W_p = 1 \mid \boldsymbol{y}, \hat{\boldsymbol{t}}, \hat{k}, cn) &= \mathrm{P}(Z_p = 1 \mid \boldsymbol{y}, \hat{\boldsymbol{t}}, \hat{k}, cn) \\
&= \frac{p(\boldsymbol{y}_{\hat{t}_{p-1},\hat{t}_p} \mid Z_p = 1, \hat{\boldsymbol{t}}, \hat{k}, cn)\mathrm{P}(Z_p = 1 \mid \hat{\boldsymbol{t}}, \hat{k}, cn)}{p(\boldsymbol{y}_{\hat{t}_{p-1},\hat{t}_p} \mid \hat{\boldsymbol{t}}, \hat{k}, cn)} \\
&= \frac{\mathrm{P}(\boldsymbol{y}_{\hat{t}_{p-1},\hat{t}_p} \mid Z_p = 0, \hat{\boldsymbol{t}}, \hat{k}, cn)\mathrm{P}(Z_p = 1 \mid \hat{\boldsymbol{t}}, \hat{k}, cn)}{\mathrm{A}^0_{\hat{t}_{p-1}\,\hat{t}_p}} \\
&= \frac{1}{\mathrm{A}^0_{\hat{t}_{p-1}\,\hat{t}_p}}\Big[p(\boldsymbol{y}_{\hat{t}_{p-1},\hat{t}_p} \mid Z_p = 0, U_p = 1, \hat{\boldsymbol{t}}, \hat{k}, cn)\mathrm{P}(U_p = 1) \\
&+ p(\boldsymbol{y}_{\hat{t}_{p-1},\hat{t}_p} \mid Z_p = 0, U_p = 0, \hat{\boldsymbol{t}}, \hat{k}, cn)\mathrm{P}(U_p = 0)\Big]\mathrm{P}(Z_p = 1 \mid \hat{\boldsymbol{t}}, \hat{k}, cn),
\end{aligned}$$

by using Equation (5), the definition of A^0_{ij}, the equality between $p(\boldsymbol{y} \mid Z_p = 0, \hat{\boldsymbol{t}}, \hat{k}, cn)$ and $p(\boldsymbol{y} \mid Z_p = 1, \hat{\boldsymbol{t}}, \hat{k}, cn)$ (given by the previous discussion) and conditioning with respect to U_p.

S.4 Algorithm to determine maxima of a multimodal function

In Section "Methods", we introduced the paired estimators ($\widehat{K}_{Peaks,thr_1,thr_2}$, $\widehat{\boldsymbol{T}}_{Peaks,thr_1,thr_2}$) for the number of segments and the breakpoints. They correspond to the number and the locations

of the peaks, respectively, of the vector $\boldsymbol{p}$ of the posterior probabilities to be a breakpoint at each SNP location. To compute them, we derived an algorithm for the determination of the maxima in a multimodal function.

Let us assume that we have to determine the positions of the maxima of a multimodal function f and we know its values at positions $\{1, \ldots, n\}$ (called $\boldsymbol{f} = \{f_1, \ldots, f_n\}$). Moreover, the values $\boldsymbol{f}$ are affected by noise (in fact, in our case f is the posterior probability to be a breakpoint at each position, which depends on the estimates of the parameters).

In this framework, we have derived an algorithm to determine the positions of the maxima of f:

1. **Denoising of $\boldsymbol{f}$.** In order to denoise the function, we use a regression method with kernel basis, obtaining $\hat{\boldsymbol{f}}$.

2. **Selection of only one position per peak.** We identify the positions which belong to the same peak through a threshold thr_1 (i.e. an interval A corresponds to a peak if all elements of $\hat{\boldsymbol{f}}_A$ are greater than thr_1). Then, among the positions belonging to the same peak, we select the one with the highest value of $\hat{f}$. The vector of guess locations is called $\boldsymbol{q}^0$.

3. **Final selection of the peak locations.** In the end, we choose all locations $i \in \boldsymbol{q}^0$ such that $\hat{f}_i > thr_2$. The new vector of locations is denoted by $\boldsymbol{q}^1$. The use of a second threshold is necessary, because the function f (i.e. the estimated posterior probabilities, in our case) can have small peaks also when it assumes values very close to zero (due to the noise).

   Moreover, since we cannot estimate more than k_{max} breakpoints (because of the definition of the prior of K), if more than k_{max} peaks are selected, then the algorithm chooses the ones corresponding to the k_{max} highest values of the set $\{\hat{f}_i \mid i \in \boldsymbol{q}^1\}$.

The described algorithm depends on the value of the thresholds thr_1 and thr_2. In the simulations in Section "Results and Discussion", we try several pairs of the following types of thresholds:

- "005" := $max(0.005, \text{quantile of } \boldsymbol{p} \text{ at } 0.95)$
- "01" := $max(0.01, \text{quantile of } \boldsymbol{p} \text{ at } 0.95)$
- "01_90" := $max(0.01, \text{quantile of } \boldsymbol{p} \text{ at } 0.90)$
- "mad" := $median(\boldsymbol{p}) + 3 * mad(\boldsymbol{p})$

where mad is the median absolute deviation. All these thresholds derive from different definitions of which probability values are to be considered significant.

S.5 Comparison among the breakpoint estimators on simulated data

As explained in Section "Results and Discussion", we compared the boundary estimators described in Section "Methods", by applying them on two artificial datasets (called datasets A and B), each of 100 samples. The boundary estimators considered were: $\widehat{\boldsymbol{T}}_{BinErrAk}$, $\widehat{\boldsymbol{T}}_{Joint}$ and $\widehat{\boldsymbol{T}}_{Peaks,thr_1,thr_2}$, for several pairs of thresholds thr_1 and thr_2 chosen among the following ones:

- "005" $:= max(0.005, \text{quantile of } \boldsymbol{p} \text{ at } 0.95)$
- "01" $:= max(0.01, \text{quantile of } \boldsymbol{p} \text{ at } 0.95)$
- "01_90" $:= max(0.01, \text{quantile of } \boldsymbol{p} \text{ at } 0.90)$
- "mad" $:= median(\boldsymbol{p}) + 3 * mad(\boldsymbol{p})$

where mad is the median absolute deviation and $\mathbf{p}$ is the vector of posterior probabilities to be a breakpoint. How we simulated the two datasets has been already explained in Section "Results and Discussion".

S.5.1 Description of the error measures

To evaluate the performance of the estimators, we used several error measures. For the estimation of the number of segments, we considered the following errors:

$$\begin{aligned} \text{0-1 error} &= 1 - \delta_{\hat{k},k_0} \\ \text{absolute error} &= |\hat{k} - k_0| \\ \text{squared error} &= (\hat{k} - k_0)^2. \end{aligned}$$

For the evaluation of the boundary estimation, we computed the binary error, i.e.

$$k_0 - 1 - \sum_{q=1}^{\hat{k}-1} \sum_{p=1}^{k_0-1} \delta_{\hat{t}_q, t_p^0},$$

the sensitivity (proportion of true breakpoints detected) and the false discovery rate (FDR, i.e. proportion of false estimated breakpoints among the estimated ones). The last two measures were calculated not only looking at the exact position of the breakpoints (w = 0), but also accounting for a neighborhood of up to 6 SNPs around the true position (w = 1, . . ., 6). Finally, to asses the influence of the boundary estimation on the profile estimation, we calculated the sum of squared distance (SSQ) and the sum 0-1 error, which are defined as

$$\begin{aligned} \text{SSQ} &= \sum_{i=1}^{n} (\widehat{\widetilde{Z}}_i - \widetilde{Z}_i^0)^2 \\ \text{sum 0-1 error} &= \sum_{i=1}^{n} \left(1 - \delta_{\widehat{\widetilde{Z}}_i, \widetilde{Z}_i^0}\right). \end{aligned}$$

We also measured the sensitivity and the FDR for all copy number events.

To compare the estimators, we considered not only the error measures computed on their estimates but also on their "final" estimates (denoted by "final" or F). In fact, since the levels are categorical variables and they are estimated separately (see Equation (4)), if the estimated levels of contiguous segments are the same, then they are joined together ("merging" step). Therefore, after the "merging", the number of the segments can be lower than the estimated one.

S.5.2 Results of the comparisons

We applied the following pairs of estimators to dataset A: $(\widehat{K}_{01}, \widehat{\boldsymbol{T}}_{BinErrAk})$, $(\widehat{K}_{01}, \widehat{\boldsymbol{T}}_{Joint})$ and $(\widehat{K}_{Peaks,005,005}, \widehat{\boldsymbol{T}}_{Peaks,005,005})$. We found that $(\widehat{K}_{01}, \widehat{\boldsymbol{T}}_{BinErrAk})$ and $(\widehat{K}_{Peaks,005,005}, \widehat{\boldsymbol{T}}_{Peaks,005,005})$ were the best performing methods. In particular, the former had the lowest binary error, regarding both the estimated boundaries and the "final" ones (see Supplementary Table S.2) and the lowest "final" FDR (see Supplementary Figure S.1), while the errors regarding the "final" estimation of the number of segments were similar (see Supplementary Table S.3). As a consequence, regarding the level estimation, $(\widehat{K}_{01}, \widehat{\boldsymbol{T}}_{BinErrAk})$ had the lowest errors (see Table 3) and almost always the highest sensitivity and lowest FDR (see Supplementary Tables S.4 and S.5).

From these results, we decided to not apply the estimators $(\widehat{K}_{01}, \widehat{\boldsymbol{T}}_{Joint})$ on dataset B and we also decided to try other paired thresholds for $\widehat{\boldsymbol{T}}_{Peaks,thr_1,thr_2}$, in order to reduce the FDR of the boundary estimation. The results showed that the methods which obtained a better estimation of the number of segments were, in order: $(\widehat{K}_{Peaks,mad,01}, \widehat{\boldsymbol{T}}_{Peaks,mad,01})$, $(\widehat{K}_{01}, \widehat{\boldsymbol{T}}_{BinErrAk})$, $(\widehat{K}_{Peaks,01,01}, \widehat{\boldsymbol{T}}_{Peaks,01,01})$ and $(\widehat{K}_{Peaks,005,005}, \widehat{\boldsymbol{T}}_{Peaks,005,005})$; see Supplementary Table S.3. Instead, regarding the boundary estimation, the methods with the lowest binary error were: $(\widehat{K}_{01}, \widehat{\boldsymbol{T}}_{BinErrAk})$, $(\widehat{K}_{Peaks,01,mad}, \widehat{\boldsymbol{T}}_{Peaks,01,mad})$, $(\widehat{K}_{Peaks,01,01}, \widehat{\boldsymbol{T}}_{Peaks,01,01})$ and $(\widehat{K}_{Peaks,005,005}, \widehat{\boldsymbol{T}}_{Peaks,005,005})$; see Supplementary Table S.2.

In general, the methods $(\widehat{K}_{Peaks,01,01}, \widehat{\boldsymbol{T}}_{Peaks,01,01})$ and $(\widehat{K}_{Peaks,005,005}, \widehat{\boldsymbol{T}}_{Peaks,005,005})$ always obtained similar results and the latter perform slightly worse than the former (e.g. in the estimation of k_0). Moreover, the methods $(\widehat{K}_{Peaks,01\_90,01\_90}, \widehat{\boldsymbol{T}}_{Peaks,01\_90,01\_90})$ and $(\widehat{K}_{Peaks,mad,mad}, \widehat{\boldsymbol{T}}_{Peaks,mad,mad})$ had always all the error measures higher than $(\widehat{K}_{Peaks,01,01}, \widehat{\boldsymbol{T}}_{Peaks,01,01})$. Therefore, the pair of estimators $(\widehat{K}_{Peaks,005,005}, \widehat{\boldsymbol{T}}_{Peaks,005,005})$, $(\widehat{K}_{Peaks,01\_90,01\_90}, \widehat{\boldsymbol{T}}_{Peaks,01\_90,01\_90})$ and $(\widehat{K}_{Peaks,mad,mad}, \widehat{\boldsymbol{T}}_{Peaks,mad,mad})$ will not be considered in the following discussions.

The lowest errors in the estimation of the number of the segments were achieved by $(\widehat{K}_{Peaks,mad,01}, \widehat{\boldsymbol{T}}_{Peaks,mad,01})$; see Supplemantary Table S.3. Moreover, using this procedure, k_0 was underestimated in about half of the cases and thus also the FDR regarding the boundary estimation was the lowest one (see Supplementary Figure S.2). As a consequence, all the error measures regarding the level estimation were among the best ones (see Table 3 and Supplementary Tables S.4 and S.5). Instead, using $(\widehat{K}_{Peaks,01,mad}, \widehat{\boldsymbol{T}}_{Peaks,01,mad})$, the number of segments was almost always overestimated (see Supplementary Table S.3) and thus the algorithm detected the highest number of true breakpoints (in fact, it had the highest sensitivity, in Supplemetary Figure S.3, and a low binary error, in Supplementary Table S.2). But due to the higher number of segments, the algorithm found also a higher number of false breakpoints than $(\widehat{K}_{Peaks,mad,01}, \widehat{\boldsymbol{T}}_{Peaks,mad,01})$ (see Supplementary Figure S.2).

From the study of the behavior of $(\widehat{K}_{Peaks,mad,01}, \widehat{\boldsymbol{T}}_{Peaks,mad,01})$ and $(\widehat{K}_{Peaks,01,mad}, \widehat{\boldsymbol{T}}_{Peaks,01,mad})$, we can understand the role of the two thresholds in our algorithm for the determination of the maxima in a multimodal function (see Section S.4). The threshold thr_1 is used to decide which points belong to the same peak: all the points, between two regions of points below thr_1, are considered in the same peak. Hence, with a low threshold, more points are considered belonging to the same peak and thus we can eliminate lot of false breakpoints (like in $(\widehat{K}_{Peaks,mad,01}, \widehat{\boldsymbol{T}}_{Peaks,mad,01})$). But, at the same time, if two true peaks are close, then it is possible that they are considered as only one peak, losing a true breakpoint (low sensitivity). Instead, the threshold thr_2 is used to choose which estimated breakpoints are significant for the regression, i.e. if their posterior probabilities are to be considered different from zero. Therefore, using a lower value of thr_2, we select a higher number of breakpoints obtaining a higher percentage of both false ones (high FDR) and true ones (high sensitivity, as in $(\widehat{K}_{Peaks,01,mad}, \widehat{\boldsymbol{T}}_{Peaks,01,mad})$).

In conclusion, from these results we suggest the use of the following pairs of estimators: $(\widehat{K}_{Peaks,01,01}, \widehat{\boldsymbol{T}}_{Peaks,01,01})$, $(\widehat{K}_{Peaks,01,mad}, \widehat{\boldsymbol{T}}_{Peaks,01,mad})$ or $(\widehat{K}_{Peaks,mad,01}, \widehat{\boldsymbol{T}}_{Peaks,mad,01})$.

S.6 Results of the comparisons on simulated data with LOH regions

As explained in Section "Results and Discussion", in order to evaluate the IBD/UPD detection of gBPCR, we compared the estimation of gBPCR with the ones given by three well-known methods in the field (dChip [16], CNAT 4.01 [36] and PennCNV [24]) using 18 simulated samples of [35]. The evaluation has been done computing the true positive rate (TPR) and the false positive rate (FPR), i.e. the proportion of SNPs inside the LOH regions that are correctly identified (as belonging to a LOH region) and the proportion of SNPs outside these segments that are wrongly identified (as belonging to them), respectively. We used $(\widehat{K}_{Peaks,01,01}, \widehat{\boldsymbol{T}}_{Peaks,01,01})$, $(\widehat{K}_{Peaks,01,mad}, \widehat{\boldsymbol{T}}_{Peaks,01,mad})$ or $(\widehat{K}_{Peaks,mad,01}, \widehat{\boldsymbol{T}}_{Peaks,mad,01})$ as paired estimators of the number of segments and the boundaries, and either $p_{upd} = 10^{-3}$ or $p_{upd} = 10^{-4}$ as the prior probability of IBD/UPD.

Since CNAT does not consider the *NoCall* SNPs (called **non-informative** SNPs) for the estimation of the LOH profile, first we compare the TPR and FPR computed using only the informative SNPs.

Regarding the IBD/UPD detection (see Supplementary Figure S.4), all methods maintained a similar and low FPR (usually below 0.2), for all samples and all SNRs. The TPR of CNAT and all versions of gBPCR was always closed to one, while dChip achieved a lower TPR (about 0.9) in the samples with SNR = 1.25. On the contrary, PennCNV always gave a TPR and a FPR close to zero, showing that it was not able to detect any IBD/UPD region.

In the estimation of losses (see Supplementary Figure S.5), we observed that, again, the FPR was always below 0.2. In case of dChip and CNAT, we saw that the FPR decreased as the noise increased, while the opposite occured for the versions of gBPCR. It is natural to observe an increasing of the FPR with the noise, because the higher the noise, the more difficult it is to perform the estimation. Therefore, the unnatural behavior of the FPR of dChip and CNAT is related to the fact that they lose in "power of detection" in presence of high noise. In fact, also their TPR decreased as the noise increased and dChip even achieved a TPR close to zero

in the samples with SNR = 1.25. PennCNV gave a TPR close to 0.2 in the samples with SNR = 5 and a TPR close to zero in the other samples. Only gBPCR maintained a TPR always close to one.

Using only the non-informative SNPs, we obtained that the TPRs, regarding the estimation of both the copy-neutral LOH and the loss, were similar to the ones previously described, for both dChip and all versions of gBPCR (see Supplementary Figures S.6 and S.7). The FPR of dChip never exceeded 0.06 and the one of gBPCR was usually lower than 0.2. Moreover, among the versions of gBPCR, ($\widehat{K}_{Peaks,01,01}$, $\widehat{\boldsymbol{T}}_{Peaks,01,01}$) and ($\widehat{K}_{Peaks,01,mad}$, $\widehat{\boldsymbol{T}}_{Peaks,01,mad}$) with $p_{upd} = 10^{-4}$ more often achieved a lower FDR than the others.

Overall, all versions of gBPCR behaved similarly on these data and they outperformed PennCNV, CNAT and dChip. Moreover, we observed that dChip failed to give a good estimation in presence of high noise, while PennCNV did not detect almost any LOH aberration. Due to the results obtained on the non-informative SNPs, we suggest to use ($\widehat{K}_{Peaks,01,01}$, $\widehat{\boldsymbol{T}}_{Peaks,01,01}$) or ($\widehat{K}_{Peaks,01,mad}$, $\widehat{\boldsymbol{T}}_{Peaks,01,mad}$) with $p_{upd} = 10^{-4}$.

S.7 Supplementary tables

	segment														
	I	II	III	IV	V	VI	VII	VIII	IX	X	XI	XII	XIII	XIV	XV
$P(Z_p = 2)$	0	0	0	0	0.8	0	0	0	0	0.8	0	0	0.8	0	0
$P(Z_p = 0)$	0.1	0.8	0.1	0.8	0.2	0.8	0.1	0	0.8	0.2	0.1	0.8	0.2	0.8	0.1
$P(Z_p = -1)$	0.8	0.2	0.8	0.2	0	0.2	0.8	0.2	0.2	0	0.8	0.2	0	0.2	0.8
$P(Z_p = -2)$	0.1	0	0.1	0	0	0	0.1	0.8	0	0	0.1	0	0	0	0.1

Table S.1: Prior distribution of $\boldsymbol{Z}$ in the simulated dataset A.

dataset	method	type	binary error
A	$(\widehat{K}_{01},\ \widehat{\boldsymbol{T}}_{BinErrAk})$	estimated	4.19
		final	4.43
	$(\widehat{K}_{01},\ \widehat{\boldsymbol{T}}_{Joint})$	estimated	7.23
		final	7.23
	$(\widehat{K}_{Peaks,005,005},\ \widehat{\boldsymbol{T}}_{Peaks,005,005})$	estimated	6.09
		final	6.16
B	$(\widehat{K}_{01},\ \widehat{\boldsymbol{T}}_{BinErrAk})$	estimated	6.53
		final	7.37
	$(\widehat{K}_{Peaks,005,005},\ \widehat{\boldsymbol{T}}_{Peaks,005,005})$	estimated	6.97
		final	7.33
	$(\widehat{K}_{Peaks,01,01},\ \widehat{\boldsymbol{T}}_{Peaks,01,01})$	estimated	6.97
		final	7.33
	$(\widehat{K}_{Peaks,01\_90,01\_90},\ \widehat{\boldsymbol{T}}_{Peaks,01\_90,01\_90})$	estimated	7.21
		final	7.46
	$(\widehat{K}_{Peaks,mad,mad},\ \widehat{\boldsymbol{T}}_{Peaks,mad,mad})$	estimated	7.40
		final	7.51
	$(\widehat{K}_{Peaks,01,mad},\ \widehat{\boldsymbol{T}}_{Peaks,01,mad})$	estimated	6.59
		final	7.00
	$(\widehat{K}_{Peaks,mad,01},\ \widehat{\boldsymbol{T}}_{Peaks,mad,01})$	estimated	7.53
		final	7.64

Table S.2: Binary error of the boundary estimations obtained with several boundary estimators on datasets A and B. On the former dataset $(\widehat{K}_{01},\ \widehat{\boldsymbol{T}}_{BinErrAk})$ outperforms all other methods, while on the latter $(\widehat{K}_{Peaks,01,mad},\ \widehat{\boldsymbol{T}}_{Peaks,01,mad})$, $(\widehat{K}_{01},\ \widehat{\boldsymbol{T}}_{BinErrAk})$, $(\widehat{K}_{Peaks,01,01},\ \widehat{\boldsymbol{T}}_{Peaks,01,01})$ and $(\widehat{K}_{Peaks,005,005},\ \widehat{\boldsymbol{T}}_{Peaks,005,005})$ give the lower binary errors.

dataset	method	type	err 0-1	err 1	err 2	$\#(\hat{k} > k_0)$
A	$(\widehat{K}_{01}, \widehat{\boldsymbol{T}}_{BinErrAk})$	estimated	1	15.74	256.2	100
		final	0.85	1.73	4.35	20
	$(\widehat{K}_{01}, \widehat{\boldsymbol{T}}_{Joint})$	estimated	1	15.74	256.2	100
		final	1	15.69	254.51	100
	$(\widehat{K}_{Peaks,005,005}, \widehat{\boldsymbol{T}}_{Peaks,005,005})$	estimated	0.99	7.43	65.55	99
		final	0.84	2.33	9.55	65
B	$(\widehat{K}_{01}, \widehat{\boldsymbol{T}}_{BinErrAk})$	estimated	0.98	6.61	57.29	96
		final	0.89	2.46	9.22	16
	$(\widehat{K}_{Peaks,005,005}, \widehat{\boldsymbol{T}}_{Peaks,005,005})$	estimated	1	12.72	177.22	100
		final	0.92	3.75	20.31	82
	$(\widehat{K}_{Peaks,01,01}, \widehat{\boldsymbol{T}}_{Peaks,01,01})$	estimated	1	12.62	175.98	100
		final	0.92	3.74	20.28	81
	$(\widehat{K}_{Peaks,01\_90,01\_90}, \widehat{\boldsymbol{T}}_{Peaks,01\_90,01\_90})$	estimated	1	15.12	246.38	100
		final	0.98	5.75	40.61	94
	$(\widehat{K}_{Peaks,mad,mad}, \widehat{\boldsymbol{T}}_{Peaks,mad,mad})$	estimated	1	17.08	300.16	100
		final	0.99	7.03	58.09	99
	$(\widehat{K}_{Peaks,01,mad}, \widehat{\boldsymbol{T}}_{Peaks,01,mad})$	estimated	1	12.62	175.98	100
		final	0.98	5.47	38.25	91
	$(\widehat{K}_{Peaks,mad,01}, \widehat{\boldsymbol{T}}_{Peaks,mad,01})$	estimated	0.9	2.98	14.3	75
		final	0.83	1.73	4.95	40

Table S.3: Error measures regarding the estimation of the number of segments on both datasets A and B. The estimations were obtained using several types of estimators. On the former dataset $(\widehat{K}_{01}, \widehat{\boldsymbol{T}}_{BinErrAk})$ and $(\widehat{K}_{Peaks,005,005}, \widehat{\boldsymbol{T}}_{Peaks,005,005})$ perform equally good, on the latter the best performing methods are $(\widehat{K}_{Peaks,mad,01}, \widehat{\boldsymbol{T}}_{Peaks,mad,01})$ and $(\widehat{K}_{01}, \widehat{\boldsymbol{T}}_{BinErrAk})$, followed by $(\widehat{K}_{Peaks,01,01}, \widehat{\boldsymbol{T}}_{Peaks,01,01})$ and $(\widehat{K}_{Peaks,005,005}, \widehat{\boldsymbol{T}}_{Peaks,005,005})$.

		sensitivity			
dataset	method	$Z = 2$	$Z = 0$	$Z = -1$	$Z = -2$
A	$(\widehat{K}_{01}, \widehat{\boldsymbol{T}}_{BinErrAk})$	0.803	0.987	0.984	0.995
	$(\widehat{K}_{01}, \widehat{\boldsymbol{T}}_{Joint})$	0.912	0.977	0.926	0.931
	$(\widehat{K}_{Peaks,005,005}, \widehat{\boldsymbol{T}}_{Peaks,005,005})$	0.849	0.985	0.963	0.961
B	$(\widehat{K}_{01}, \widehat{\boldsymbol{T}}_{BinErrAk})$	0.681	0.932	0.968	0.555
	$(\widehat{K}_{Peaks,005,005}, \widehat{\boldsymbol{T}}_{Peaks,005,005})$	0.894	0.983	0.961	0.946
	$(\widehat{K}_{Peaks,01,01}, \widehat{\boldsymbol{T}}_{Peaks,01,01})$	0.896	0.983	0.961	0.946
	$(\widehat{K}_{Peaks,01\_90,01\_90}, \widehat{\boldsymbol{T}}_{Peaks,01\_90,01\_90})$	0.884	0.981	0.940	0.930
	$(\widehat{K}_{Peaks,mad,mad}, \widehat{\boldsymbol{T}}_{Peaks,mad,mad})$	0.893	0.979	0.928	0.923
	$(\widehat{K}_{Peaks,01,mad}, \widehat{\boldsymbol{T}}_{Peaks,01,mad})$	0.896	0.983	0.961	0.946
	$(\widehat{K}_{Peaks,mad,01}, \widehat{\boldsymbol{T}}_{Peaks,mad,01})$	0.889	0.984	0.963	0.942

Table S.4: Sensitivity in the detection of each type of copy number event on datasets A and B. On dataset A, $(\widehat{K}_{01}, \widehat{\boldsymbol{T}}_{BinErrAk})$ and $(\widehat{K}_{Peaks,005,005}, \widehat{\boldsymbol{T}}_{Peaks,005,005})$ seem to have globally the highest sensitivity, while, on the latter dataset, $(\widehat{K}_{Peaks,005,005}, \widehat{\boldsymbol{T}}_{Peaks,005,005})$, $(\widehat{K}_{Peaks,01,01}, \widehat{\boldsymbol{T}}_{Peaks,01,01})$ and $(\widehat{K}_{Peaks,01,mad}, \widehat{\boldsymbol{T}}_{Peaks,01,mad})$ outperform all other methods.

		FDR			
dataset	method	$Z = 2$	$Z = 0$	$Z = -1$	$Z = -2$
A	$(\widehat{K}_{01}, \widehat{\boldsymbol{T}}_{BinErrAk})$	0.039	0.020	0.036	0.000
	$(\widehat{K}_{01}, \widehat{\boldsymbol{T}}_{Joint})$	0.232	0.110	0.027	0.002
	$(\widehat{K}_{Peaks,005,005}, \widehat{\boldsymbol{T}}_{Peaks,005,005})$	0.141	0.064	0.029	0.001
B	$(\widehat{K}_{01}, \widehat{\boldsymbol{T}}_{BinErrAk})$	0.017	0.047	0.306	0.025
	$(\widehat{K}_{Peaks,005,005}, \widehat{\boldsymbol{T}}_{Peaks,005,005})$	0.044	0.031	0.069	0.020
	$(\widehat{K}_{Peaks,01,01}, \widehat{\boldsymbol{T}}_{Peaks,01,01})$	0.043	0.031	0.068	0.020
	$(\widehat{K}_{Peaks,01\_90}, \widehat{\boldsymbol{T}}_{Peaks,01\_90})$	0.085	0.036	0.081	0.028
	$(\widehat{K}_{Peaks,mad,mad}, \widehat{\boldsymbol{T}}_{Peaks,mad,mad})$	0.106	0.041	0.079	0.034
	$(\widehat{K}_{Peaks,01,mad}, \widehat{\boldsymbol{T}}_{Peaks,01,mad})$	0.043	0.031	0.068	0.020
	$(\widehat{K}_{Peaks,mad,01}, \widehat{\boldsymbol{T}}_{Peaks,mad,01})$	0.038	0.026	0.075	0.023

Table S.5: FDR in the detection of each type of copy number event on datasets A and B. On dataset A, $(\widehat{K}_{01}, \widehat{\boldsymbol{T}}_{BinErrAk})$ and $(\widehat{K}_{Peaks,005,005}, \widehat{\boldsymbol{T}}_{Peaks,005,005})$ seem to have globally the lowest FDR, while, on the latter dataset, $(\widehat{K}_{Peaks,005,005}, \widehat{\boldsymbol{T}}_{Peaks,005,005})$, $(\widehat{K}_{Peaks,01,01}, \widehat{\boldsymbol{T}}_{Peaks,01,01})$ and $(\widehat{K}_{Peaks,01,mad}, \widehat{\boldsymbol{T}}_{Peaks,01,mad})$ outperform all other methods.

types of regions	Patient 1	Patient 2
in Sample .1 (total)	213	376
in Sample .2 (total)	337	384
in Sample .3 (total)	391	177
distinct (total)	438	438
equal (%)	22	27
overlapping (%)	52	52
validated (%)	73	79
single sample (%)	27	21
% of single sample $<$ 50 SNPs	28	62

Table S.6: Results regarding the IBD/UPD region detection, obtained on two patients of [37] using ($\hat{K}_{Peaks,01,01}$, $\hat{\boldsymbol{T}}_{Peaks,01,01}$), as paired estimators of the number of segments and the breakpoints, and $p_{upd} = 10^{-4}$, as probability of IBD/UPD event.

S.8 Supplementary figures

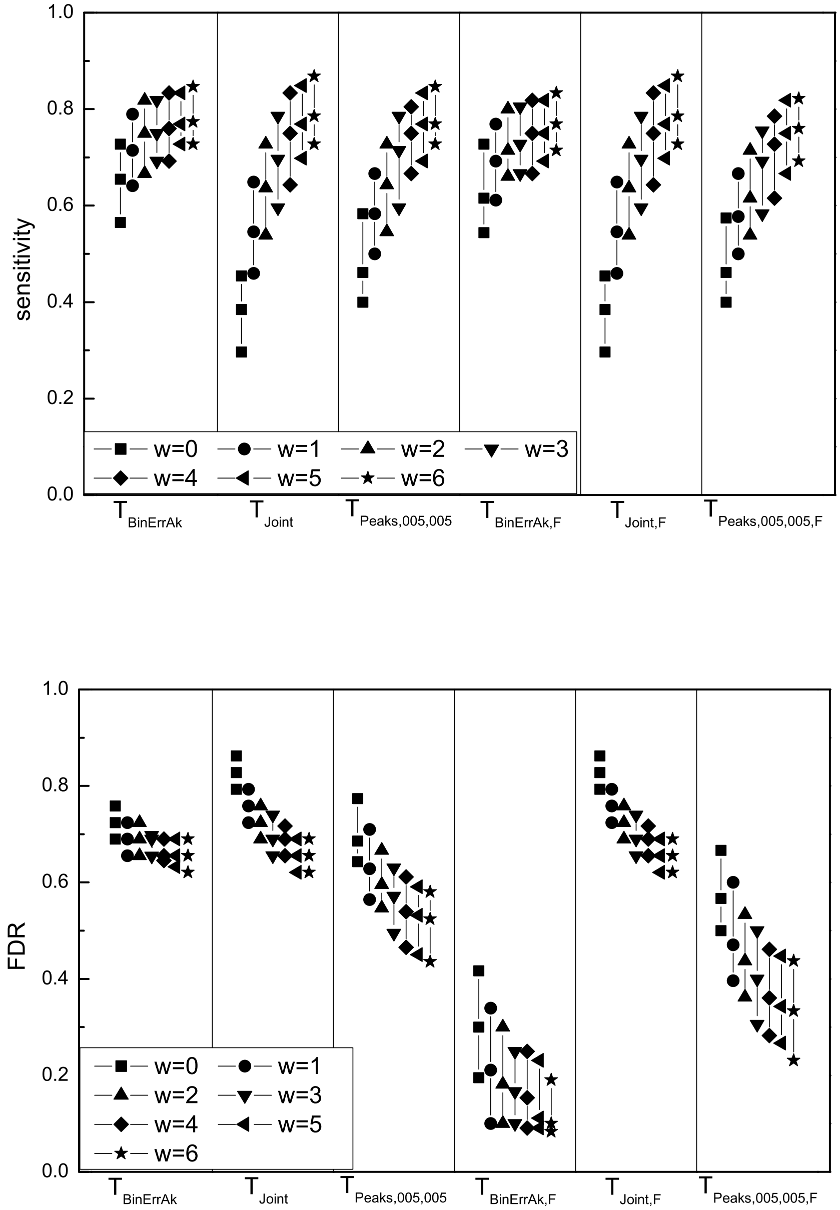

Figure S.1: Sensitivity (at the top) and FDR (at the bottom) of all boundary estimators applied to dataset A. The estimator $\widehat{\boldsymbol{T}}_{BinErrAk}$ achieves the lowest "final" FDR and $\widehat{\boldsymbol{T}}_{Joint}$ has the highest FDR. On the contrary, for $w = 6$, the sensitivities of all estimators look similar.

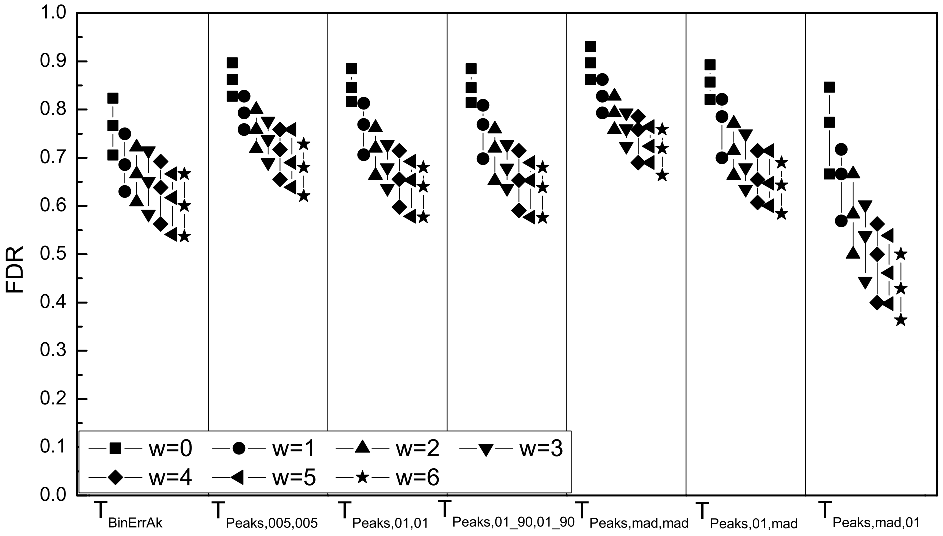

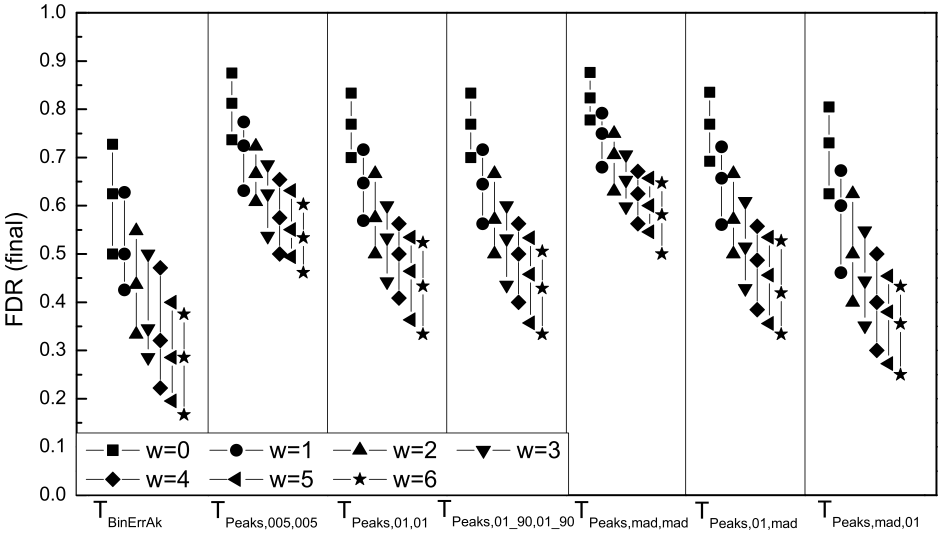

Figure S.2: FDR of all boundary estimators applied to dataset B computed on both the original estimates (at the top) and the final ones (at the bottom). The estimator $\hat{\boldsymbol{T}}_{Peaks,mad,01}$ has the lowest FDR. Instead, $\widehat{\boldsymbol{T}}_{BinErrAk}$ achieves the lowest final FDR, followed by $\hat{\boldsymbol{T}}_{Peaks,mad,01}$.

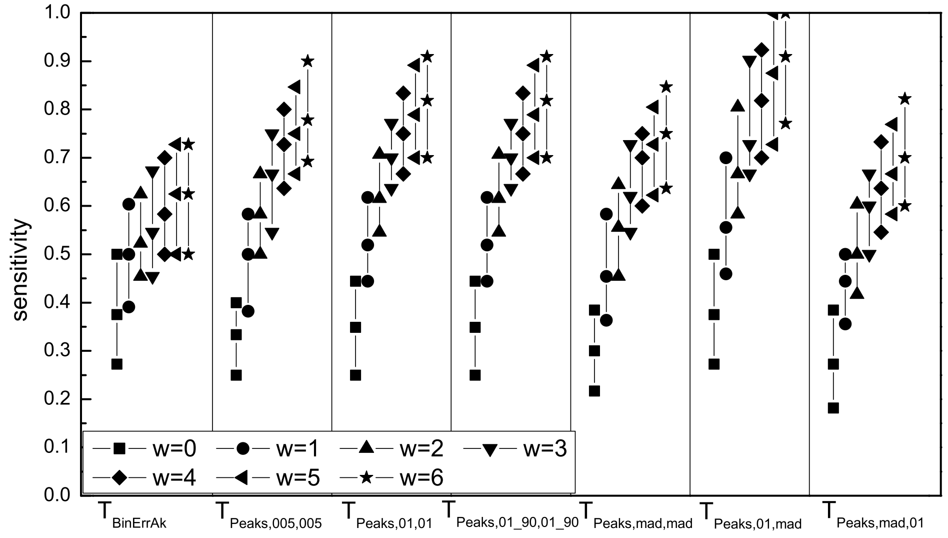

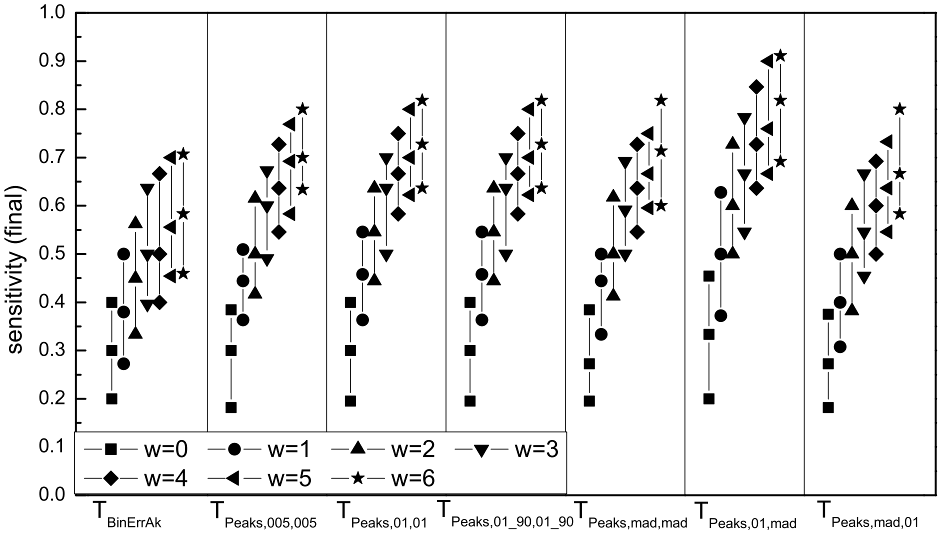

Figure S.3: Sensitivity of all boundary estimators applied to dataset B computed on both the original estimates (at the top) and the final ones (at the bottom). The estimator $\widehat{\boldsymbol{T}}_{Peaks,01,mad}$ has the highest sensitivity (and also the final one).

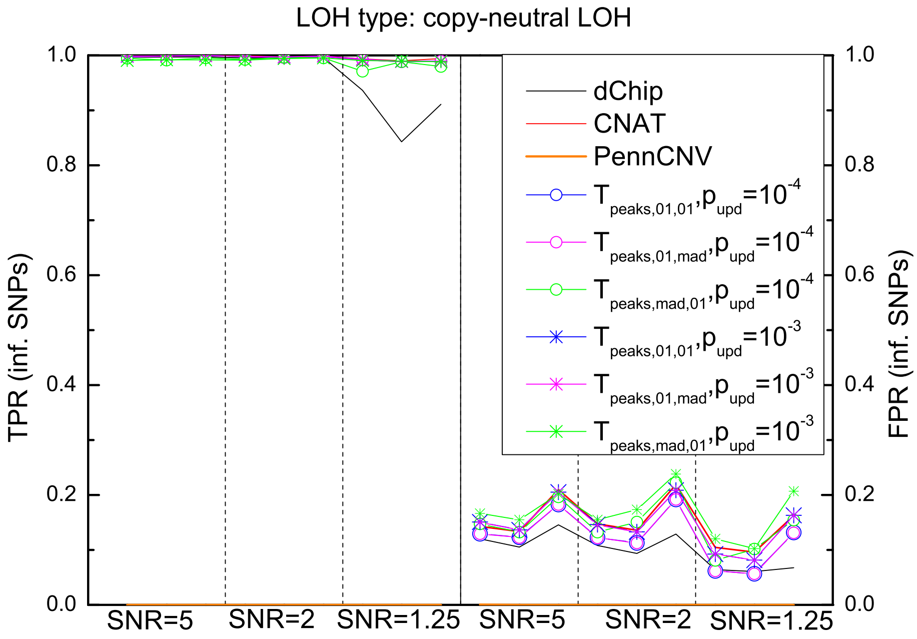

Figure S.4: TPR and FPR (computed only on informative SNP) of all methods applied to the samples, with regions of copy-neutral LOH, of the dataset in [35]. The FPR is almost always below 0.2, for all methods. All methods (apart from PennCNV) always have a TPR close to 1, but dChip shows a lower TPR in samples with SNR=1.25. The three points per SNR correspond to the three samples used.

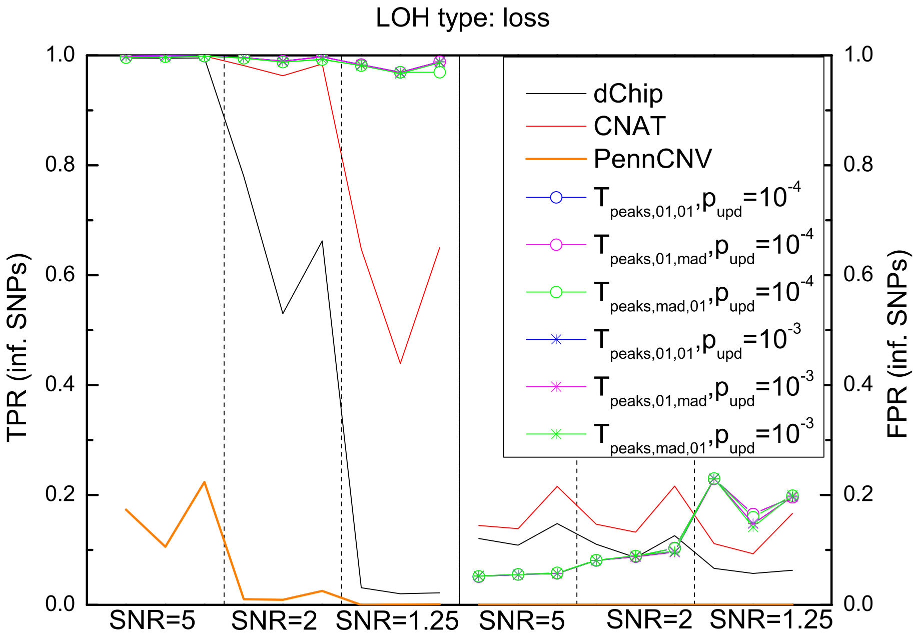

Figure S.5: TPR and FPR (computed only on informative SNP) of all methods applied to the samples, with regions of loss, of the dataset in [35]. The FPR is almost always below 0.2, for all methods. The TPR of PennCNV, dChip and CNAT decreases as the noise increases. Instead, all versions of gBPCR always maintain a TPR close to 1. The three points per SNR correspond to the three samples used.

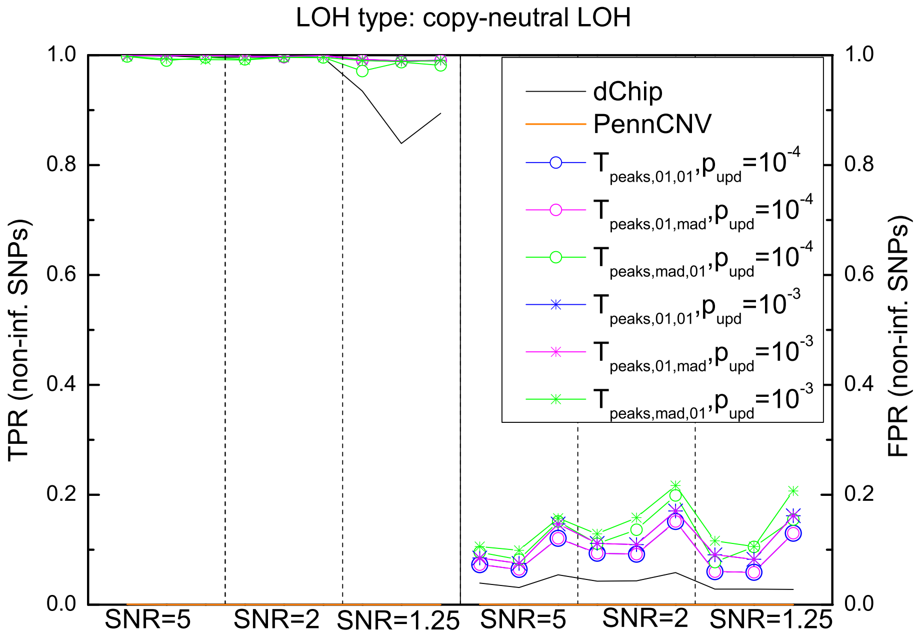

Figure S.6: TPR and FPR (computed only on non-informative SNP) of all methods applied to the samples, with regions of copy-neutral LOH, of the dataset in [35]. PennCNV achieved both a TPR and a FPR close to zero. All versions of gBPCR have a higher TPR than dChip in samples with high noise, while the FPR of dChip is usually lower than gBPCR. Among the versions of gBPCR, $(\widehat{K}_{Peaks,01,01}, \widehat{\boldsymbol{T}}_{Peaks,01,01})$ and $(\widehat{K}_{Peaks,01,mad}, \widehat{\boldsymbol{T}}_{Peaks,01,mad})$ with $p_{upd} = 10^{-4}$ more often achieve a lower FDR than the others. The three points per SNR correspond to the three samples used.

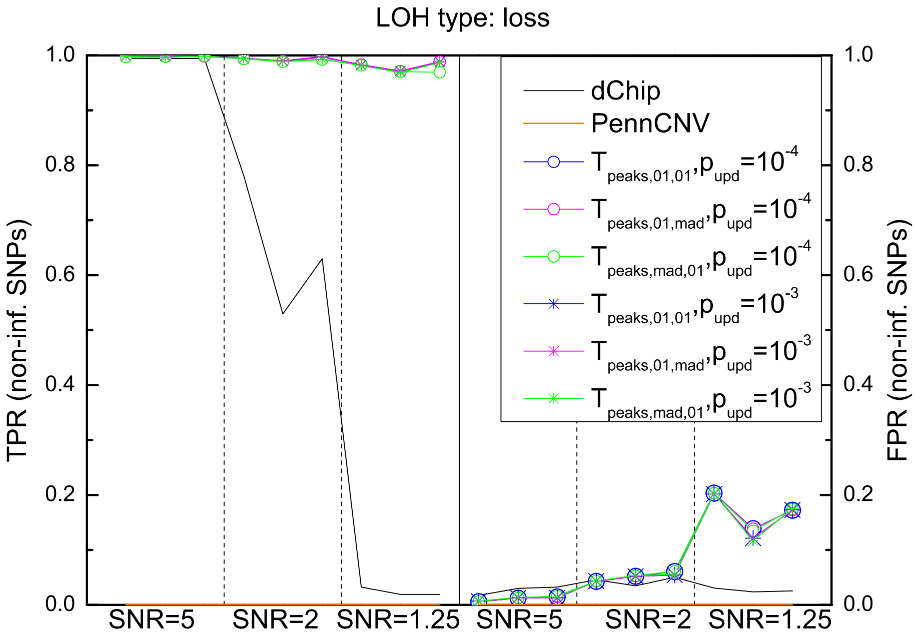

Figure S.7: TPR and FPR (computed only on non-informative SNP) of all methods applied to the samples, with regions of loss, of the dataset in [35]. For all methods, the FPR is always below 0.2. Moreover, the TPR of dChip decreases as the noise increases and the one of PennCNV is always close to zero. Instead, the TPR of all versions of gBPCR is always close to 1. The three points per SNR correspond to the three samples used.

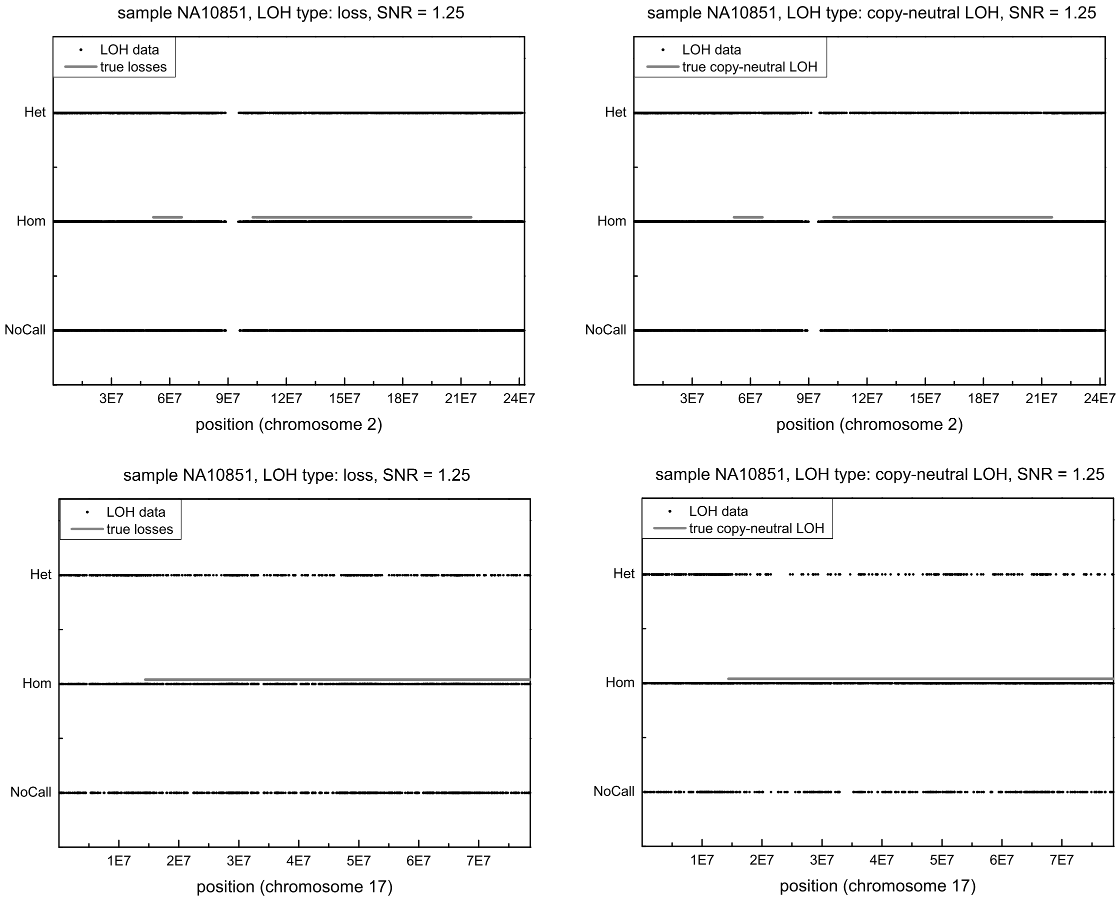

Figure S.8: LOH data of the four examples shown in Figure 6.

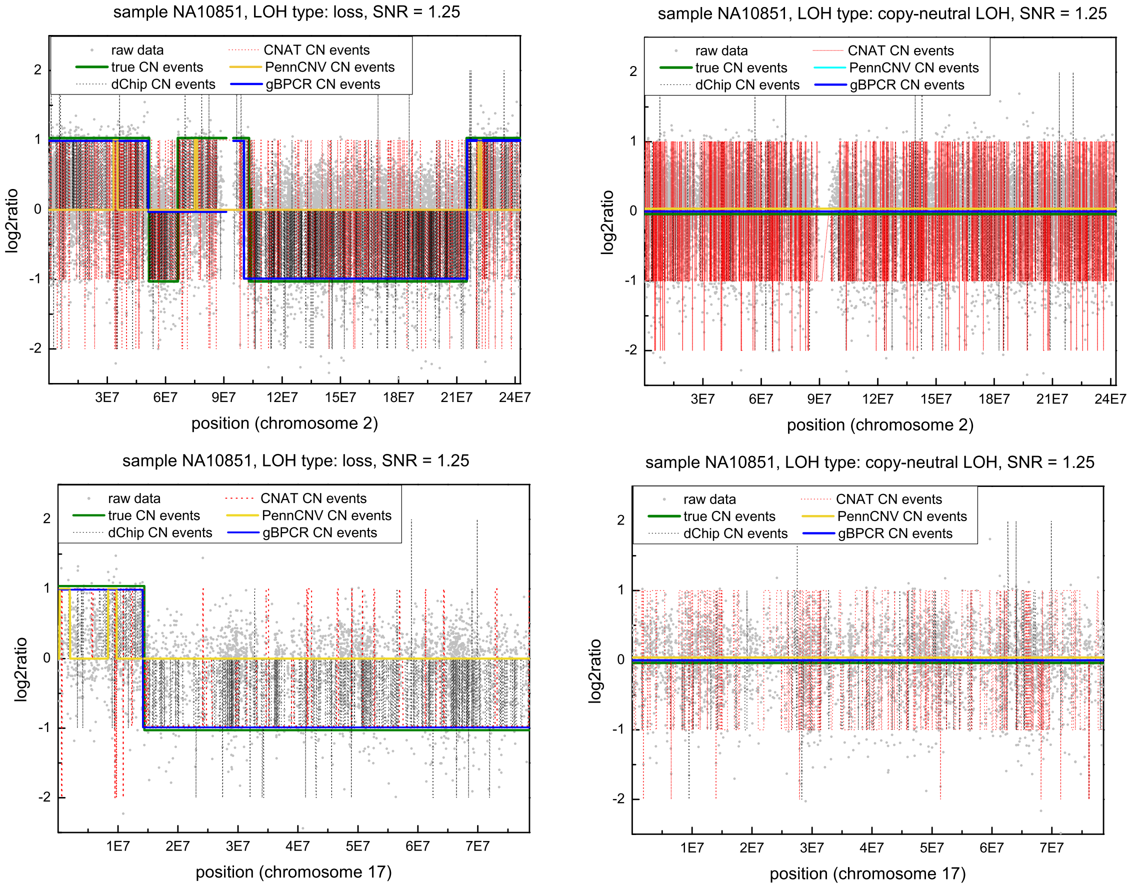

Figure S.9: Copy number event profiles of the four examples shown in Figure 6, estimated by gBPCR, PennCNV and the HMM methods implemented in dChip and CNAT. The last two procedures obtained oscillating profiles, due to the noise of the samples.

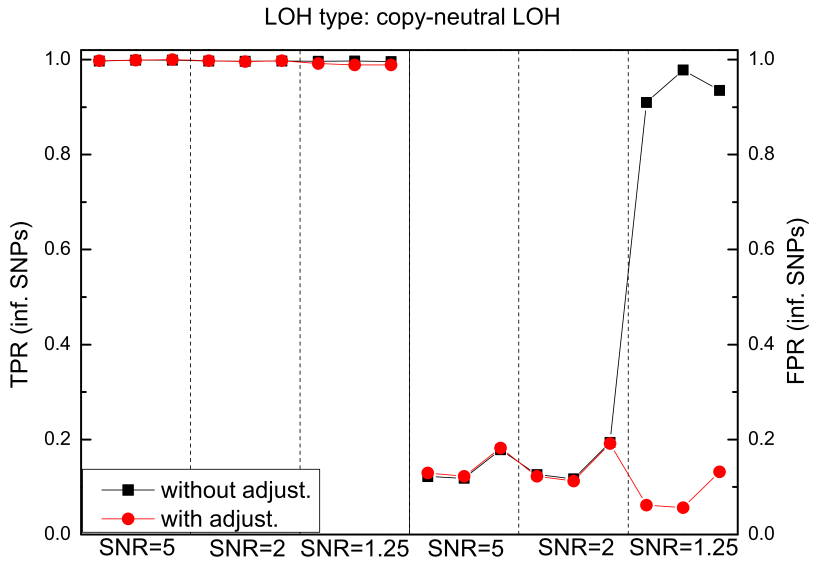

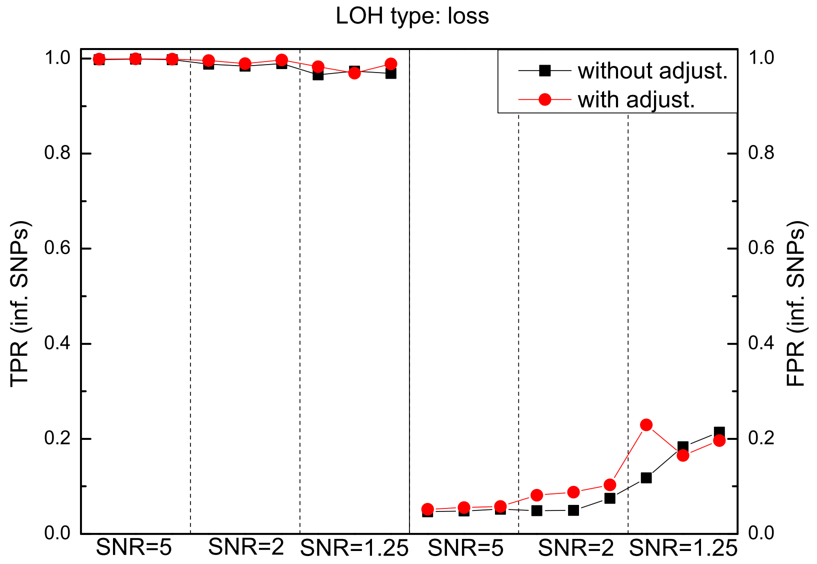

Figure S.10: TPR and FPR (computed only on informative SNP) of the versions of gBPCR, which use $(\hat{K}_{Peaks,01,01}, \hat{\boldsymbol{T}}_{Peaks,01,01})$, $p_{upd} = 10^{-4}$ and with or without the adjustment of the parameters, applied to the samples of the dataset in [35]. In case of high noise, the version without the correction has a FPR close to 1, in the detection of copy-neutral LOH regions. The three points per SNR correspond to the three samples used.

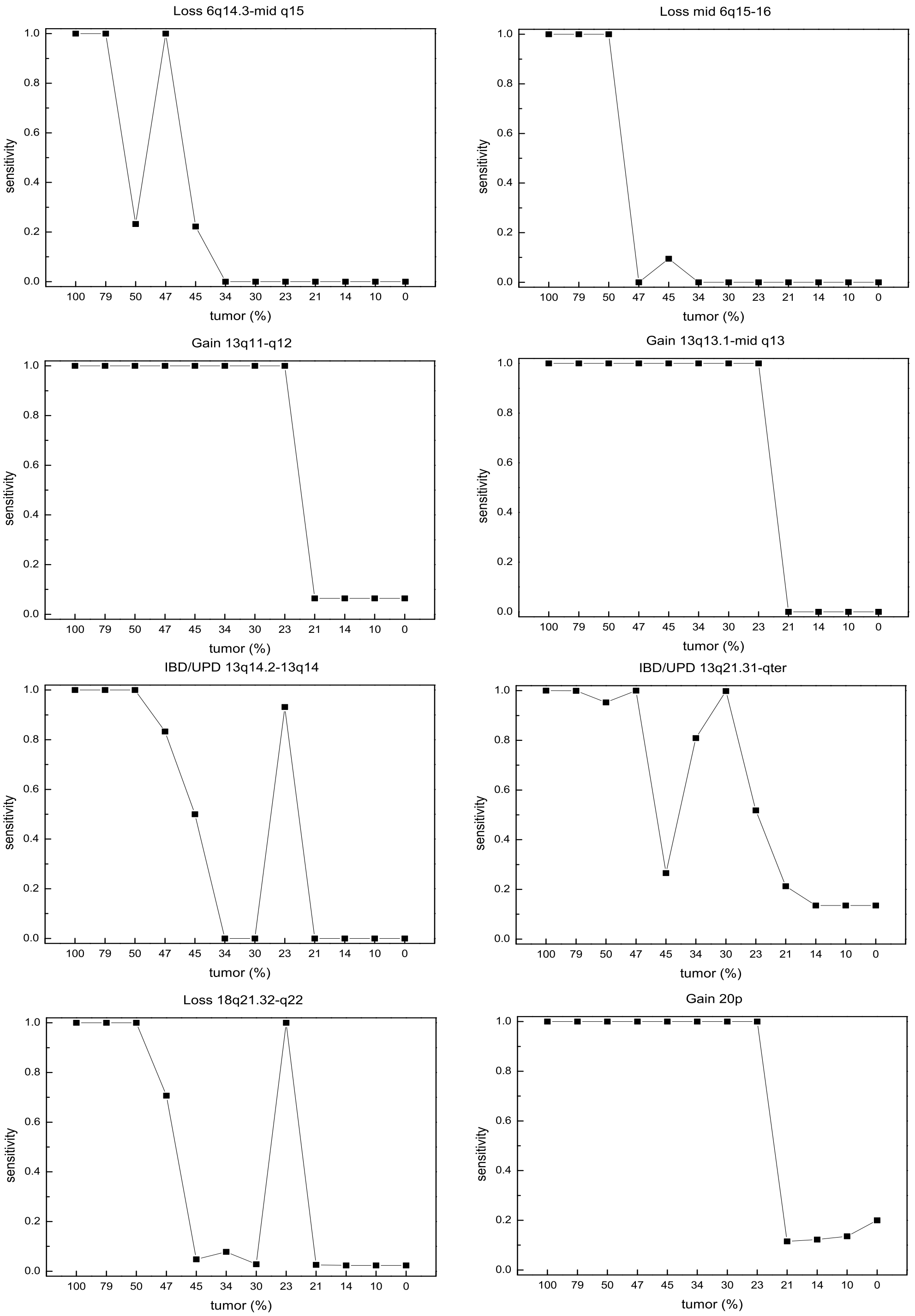

Figure S.11: Sensitivity of the version of gBPCR, which uses ($\hat{K}_{Peaks,01,01}$, $\hat{\boldsymbol{T}}_{Peaks,01,01}$) and $p_{upd} = 10^{-4}$, applied to samples of dataset in [23]. The sensitivity was calculated by looking if the algorithm found any aberration in the region considered. Comparing the results with the ones obtained by other methods (see Figure 7 in [23]), we can observe that gBPCR outperforms dChip and PennCNV and often also QuantiSNP. Sometimes gBPCR has a non-zero sensitivity in the normal sample, because it detects small IBD/UPD regions.

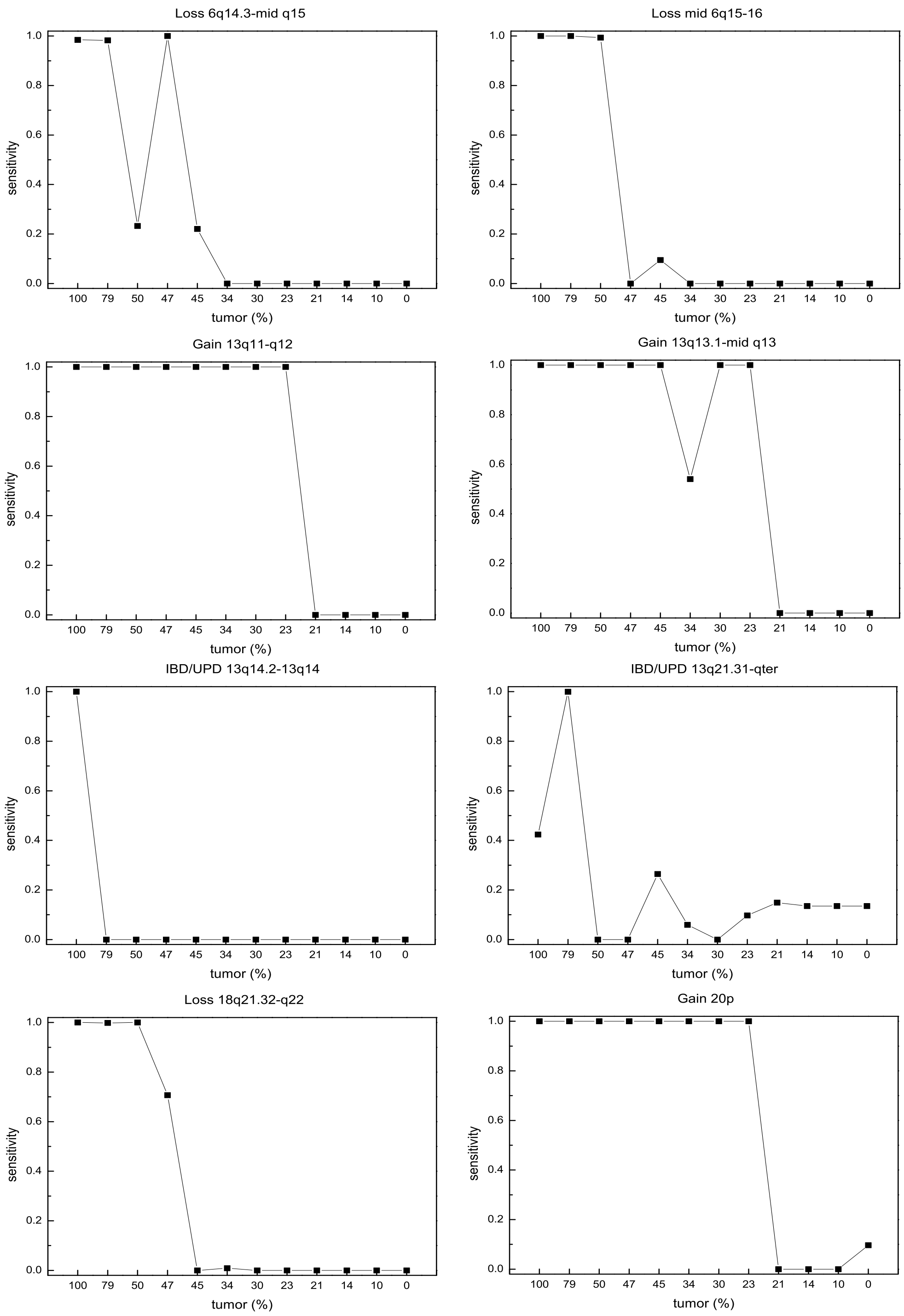

Figure S.12: Sensitivity of the version of gBPCR, which uses $(\hat{K}_{Peaks,01,01}, \hat{\boldsymbol{T}}_{Peaks,01,01})$ and $p_{upd} = 10^{-4}$, applied to samples of dataset in [23]. The sensitivity was calculated by looking if the algorithm found the correct aberration in the region considered. The algorithm gBPCR usually detects correctly the aberrations in samples with at least 60% of tumor content and its sensitivity is often higher than the one of dChip and PennCNV (see Figure 7 in [23]).

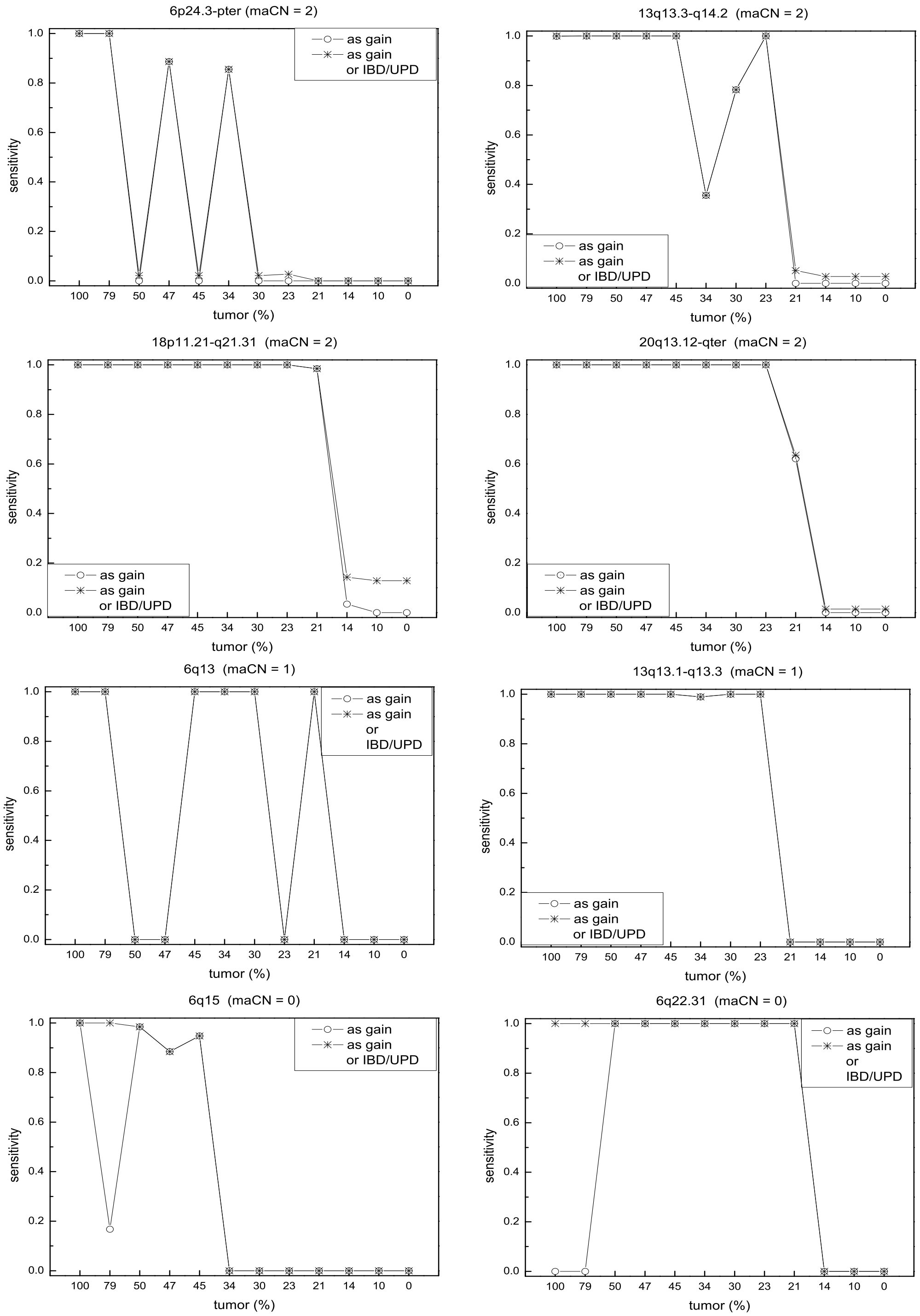

Figure S.13: Sensitivity of the version of gBPCR, which uses ($\hat{K}_{Peaks,01,01}$, $\hat{\boldsymbol{T}}_{Peaks,01,01}$) and $p_{upd} = 10^{-4}$, applied to samples of dataset in [23]. The sensitivity was calculated in two ways: by looking if the algorithm found a gain in the region considered and by looking if it found either a gain or an IBD/UPD. In few cases gBPCR estimated at least part of the gain region as an IBD/UPD, due to a high percentage of homozygous SNPs and a copy number close to the normal value.
